# The auxin-inducible degron 2 technology provides sharp degradation control in yeast, mammalian cells, and mice

**DOI:** 10.1038/s41467-020-19532-z

**Published:** 2020-11-11

**Authors:** Aisha Yesbolatova, Yuichiro Saito, Naomi Kitamoto, Hatsune Makino-Itou, Rieko Ajima, Risako Nakano, Hirofumi Nakaoka, Kosuke Fukui, Kanae Gamo, Yusuke Tominari, Haruki Takeuchi, Yumiko Saga, Ken-ichiro Hayashi, Masato T. Kanemaki

**Affiliations:** 1 Department of Chromosome Science, National Institute of Genetics, Research Organization of Information and Systems (ROIS), Yata 1111, Mishima, Shizuoka 411-8540 Japan; 2 Department of Genetics, The Graduate University for Advanced Studies (SOKENDAI), Yata 1111, Mishima, Shizuoka 411-8540 Japan; 3 FIMECS, Inc., Muraoka-Higashi 2-26-1, Fujisawa, Kanagawa 251-0012 Japan; 4 Department of Gene Function and Phenomics, National Institute of Genetics, ROIS, Yata 1111, Mishima, Shizuoka 411-8540 Japan; 5 Laboratory of Chemical Pharmacology, Graduate School of Pharmaceutical Sciences, University of Tokyo, Bunkyo, Tokyo 113-0033 Japan; 6 Department of Genomics and Evolutionary Biology, National Institute of Genetics, ROIS, Yata 1111, Mishima, Shizuoka 411-8540 Japan; 7 Department of Cancer Genome Research, Sasaki Institute, Sasaki Foundation, Kandasurugadai 2-2, Chiyoda-ku, Tokyo 101-0062 Japan; 8 Department of Biochemistry, Okayama University of Science, Ridai-cho 1-1, Okayama, 700-0005 Japan; 9 Social Cooperation Program of Evolutional Chemical Safety Assessment System, LECSAS, Graduate School of Pharmaceutical Sciences, University of Tokyo, Bunkyo, Tokyo 113-0033 Japan; 10 Department of Biological Sciences, Graduate School of Science, The University of Tokyo, Tokyo, 113-0033 Japan

**Keywords:** Genetic techniques, Expression systems, Proteolysis

## Abstract

Protein knockdown using the auxin-inducible degron (AID) technology is useful to study protein function in living cells because it induces rapid depletion, which makes it possible to observe an immediate phenotype. However, the current AID system has two major drawbacks: leaky degradation and the requirement for a high dose of auxin. These negative features make it difficult to control precisely the expression level of a protein of interest in living cells and to apply this method to mice. Here, we overcome these problems by taking advantage of a bump-and-hole approach to establish the AID version 2 (AID2) system. AID2, which employs an OsTIR1(F74G) mutant and a ligand, 5-Ph-IAA, shows no detectable leaky degradation, requires a 670-times lower ligand concentration, and achieves even quicker degradation than the conventional AID. We demonstrate successful generation of human cell mutants for genes that were previously difficult to deal with, and show that AID2 achieves rapid target depletion not only in yeast and mammalian cells, but also in mice.

## Abstract (web summary)

Auxin-inducible degron systems can be leaky and require high doses of auxin. Here the authors establish AID2 which uses an OsTIR1 mutant and the ligand 5-Ph-IAA to overcome these problems and establish AID-mediated target depletion in mice.

## Introduction

Studies of protein function in living cells and animals are greatly assisted by the conditional depletion of a protein of interest. An ideal conditional depletion should be achieved rapidly and efficiently before the resultant phenotype is complicated or compromised by secondary effects and/or adaptation. Conditional gene knockout^1^ or siRNA-based mRNA depletion^2^ has been employed in many studies. However, these technologies are not ideal for studying highly dynamic processes, such as cell cycle, differentiation or neural activity, because of the slow rate of depletion of the protein of interest. To achieve rapid depletion, protein-knockdown systems are becoming more popular.

Proteins can be targeted for rapid degradation by the ubiquitin–proteasome pathway^3^. Protein knockdown can be achieved by recruiting a protein of interest to an E3 ubiquitin ligase. For this purpose, heterobifunctional chemical degraders^4^ (known as proteolysis-targeting chimeras or PROTACs) or antibodies (e.g., Trim-Away^5^) can be used. However, to employ these methodologies, a specific PROTAC or antibody must be developed for each protein of interest. A more general approach has been developed, which uses a small tag (called degron) that induces degradation in the presence or absence of a defined ligand, so that the level of degradation of a degron-fused protein via the ubiquitin–proteasome pathway can be rapidly controlled by ligand administration^6–11^.

We pioneered development of one of the major degron-based systems, auxin-inducible degron (AID) technology^12^. When expressed in non-plant cells, *Oryza sativa* TIR1 (OsTIR1) forms a Skp1–Cul1–F-box (SCF) E3 ligase complex with endogenous components. We identified a 7-kD degron derived from *Arabidopsis* IAA17, that we termed mini-AID (mAID)^13^. We and others showed that, in many cases, mAID-fused proteins can be degraded in human cells expressing OsTIR1 with a half-life (T1/2) of 20–40 min^12,14,15^. This AID technology has recently been used in many functional studies because of its high efficiency and rapid depletion^16–19^. However, it has two major drawbacks. First, leaky degradation of mAID-fused proteins (hereafter referred to as basal degradation) was observed even without auxin^15^. In some cases, we were not successful in fusing mAID to proteins essential for viability in cells that constitutively expressed OsTIR1. In such cases, we needed to control the expression of OsTIR1 using a conditional tetracycline-inducible promoter. Second, the required doses of indole-3-acetic acid (IAA), a natural auxin used for degradation, are relatively high (typically 100–500 µM). These have no acute short-term effect in yeast and cancer cell lines, but we noted that some cell lines showed slow growth when cultured for long-term with 500 µM IAA (data will be shown later). This could be a potential problem for applying the original AID system to stem cell lines and mice. Indeed, IAA is known to cause kidney toxicity when it is converted to indoxyl sulphate in the liver^20^. As far as we know, the AID system has not been successfully applied to adult mice. Multiple groups, including our own, recently reported a method to suppress basal degradation^21–23^, but a high dose of IAA was still used in all cases.

Here, we show AID version 2 (AID2) system that overcomes all these drawbacks of the original AID system. We showed that an OsTIR1(F74G) mutant demonstrated no detectable basal degradation and that depletion could be induced at about a 670 times lower concentration of a ligand, 5-phenyl-indole-3-acetic acid (5-Ph-IAA). Moreover, mAID-fused proteins were depleted even more rapidly than using AID. We showed that an AID2 system using the OsTIR1(F74G)–5-Ph-IAA pair allowed rapid and efficient depletion of mAID-fused proteins in yeast, mammalian cells and even in mice.

## Results

### A bump-and-hole improvement of the AID system

**Fig. 1:**
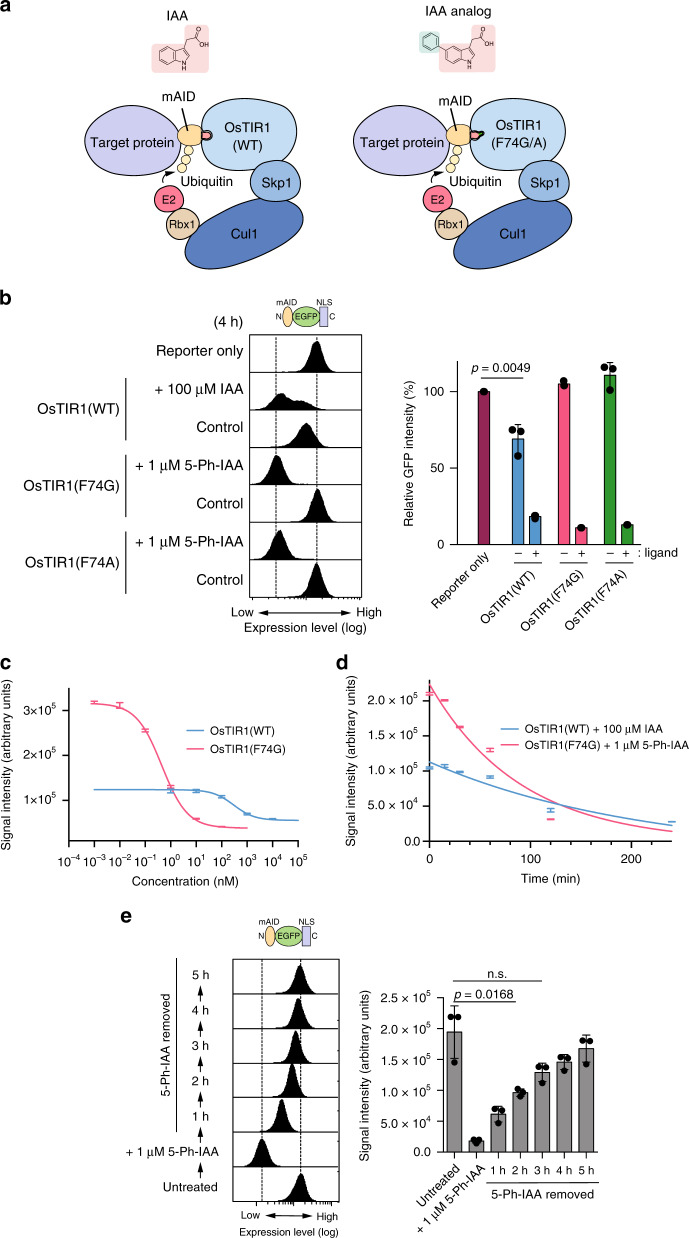
Properties of AID2 system employing the OsTIR1(F74G)–5-Ph-IAA pair. **a** Schematic illustration showing the AID and AID2 systems. **b** Depletion of an mAID-EGFP-NLS reporter in isogenic cells using the AID and AID2 systems. Indicated HCT116 cells were treated with the indicated ligand for 4 h. The graph on the right shows the quantified reporter levels relative to that of the cells expressing only the reporter. Data are presented as mean values ± SD (*n* = 3 independent experiments, two-tailed *t*-test). **c** Dose response of depletion of the reporter by IAA or 5-Ph-IAA in cells expressing OsTIR1(WT) or OsTIR1(F74G), respectively. Data are presented as mean values ± SD (*n* = 3 independent experiments). The data were fitted with non-linear regression using four parameters. **d** A time-course of depletion of the reporter induced in cells expressing OsTIR1(WT) or OsTIR1(F74G) by treating with 100 µM IAA or 1 µM 5-Ph-IAA, respectively. Data are presented as mean values ± SD (*n* = 3 independent experiments). The data were fitted with one phase decay. **e** Re-expression of the reporter after depletion by the AID2 system. HCT116 cells expressing OsTIR1(F74G) and the reporter were treated with 1 µM 5-Ph-IAA for 3 h before medium exchange. Samples were taken at the indicated time points. The graph on the right shows the quantified reporter levels. Data are presented as mean values ± SD (*n* = 3 independent experiments, two-tailed *t*-test).

Recent work reporting a bump-and-hole approach for the modification of the *Arabidopsis thaliana* TIR1 (AtTIR1)–IAA pair to hijack the auxin pathway in plants inspired us to apply a similar strategy to improving the AID technology^24^. To clarify its difference from the original AID system, we named that the AID system developed using this bump-and-hole strategy as the AID2 system. We introduced a corresponding F74G or F74A mutation in OsTIR1 to make a hole within the auxin-binding site (Fig. 1a and Supplementary Fig. 1A). We generated clones with an isogenic genotype that expressed OsTIR1(WT, F74G or F74A) together with the mAID-EGFP-NLS reporter (Supplementary Fig. 1B, C).

To find the best inducing ligand for AID2, we tested a series of bumped-IAA analogues on the reporter cells expressing OsTIR1(F74G) (Supplementary Fig. 2). Among the tested analogues, 5-Ph-IAA showed the best level of efficiency at 1 nM and 5-(3-methoxyphenyl)-indole-3-acetic acid (5-(3-MeOPh)-IAA; also known as cvxIAA) was less efficient, consistent with the previous report^24^. Therefore, we focused on 5-Ph-IAA in the subsequent experiments.

Next, we looked at side effects caused by 5-Ph-IAA treatment. HCT116 cells treated with 100 µM IAA or 1 µM 5-Ph-IAA did not show alterations in the cell cycle or in colony formation (Supplementary Fig. 3A, B). However, 500 µM IAA, a concentration commonly used for many experiments, caused alteration in colony formation (Supplementary Fig. 3B). Compared to cells treated with 100 µM IAA, we detected significantly fewer genes that showed an expression change after 1 µM 5-Ph-IAA treatment (Supplementary Fig. 3C and Supplementary Table 1). We carried out a gene ontology analysis for up- and down-regulated genes after 1 µM 5-Ph-IAA treatment and found no significant GO terms. We concluded that treatment with 1 µM 5-Ph-IAA causes less side effect compared to that with 100 µM IAA.

We compared the level of OsTIR1(WT), OsTIR1(F74G) and OsTIR1(F74A) on the isogenic backgrounds (Supplementary Fig. 1B, C) and noted that the expression level of OsTIR1(WT) was reduced compared to that of OsTIR1(F74G) and OsTIR1(F74A) (Supplementary Fig. 1D). The expression level of OsTIR1(WT) was enhanced by the addition of an OsTIR1 inhibitor auxinole^22^, suggesting that OsTIR1(WT), but not the other two mutants, was degraded by autoubiquitylation related to the basal degradation. In cells expressing OsTIR1(WT), the reporter level was low and its signal peak was broad, indicating basal degradation in the absence of IAA^15^ (Fig. 1b, left). After adding 100 µM IAA, the signal peak shifted left showing that the reporter was degraded. In sharp contrast, in cells expressing OsTIR1(F74G or F74A), the expression level was the same as in the parental cells expressing only the porter, showing that there is no basal degradation in these cells (Fig. 1b, left). Moreover, the reporter was efficiently degraded in cells expressing OsTIR1(F74G or F74A) after the addition of 1 µM 5-Ph-IAA for 4 h. OsTIR1(F74G) and OsTIR1(F74A) behaved similarly with or without the addition of 5-Ph-IAA (Fig. 1b, graph shown on the right). However, OsTIR1(F74A) was more reactive to IAA consistent with the previous report^24^ (Supplementary Fig. 1E). Therefore, we mainly used OsTIR1(F74G) in the following experiments.

To compare OsTIR1(WT) and OsTIR1(F74G) in more detail, we induced reporter degradation using a range of doses of IAA or 5-Ph-IAA, respectively (Fig. 1c). The ligand concentrations required for degradation in cells expressing OsTIR1(F74G) were significantly lower. The DC50 value (the concentration required for 50% degradation) was 300 ± 30 nM and 0.45 ± 0.01 nM for OsTIR1(WT) and OsTIR1(F74G), respectively, showing that the AID2 system using 5-Ph-IAA functioned at ~670 times lower ligand concentrations than the original AID system. To test the depletion kinetics, we took time-course samples and monitored the expression level of the reporter (Fig. 1d). We found that the AID2 system worked more rapidly, with a T1/2 of 62.3 ± 2.0 min. In contrast, the original system worked less efficiently with a T1/2 of 147.1 ± 12.5 min. Please note that the T1/2 for the reporter is longer than that for endogenous proteins because the reporter expression level is much higher. We further found that the level of OsTIR1(WT) required for efficient reporter degradation was about 1.8 times higher than OsTIR1(F74G), indicating that, at a low level, OsTIR1(F74G) works more efficiently than OsTIR1(WT) (Supplementary Fig. 1f). Taken together, the results shown in Fig. 1b–d allowed us to conclude that the AID2 system using OsTIR1(F74G) showed no detectable basal degradation, worked at significantly lower concentrations of the activating ligand, 5-Ph-IAA, and achieved faster target depletion than the AID system.

An advantage of the AID system is its reversibility^12^. To test the reversibility of the AID2 system using the OsTIR1(F74G)–5-Ph-IAA pair, we induced degradation of the reporter then replaced the culture medium with fresh medium without 5-Ph-IAA. We found that the reporter expression was mostly recovered after 3 h (Fig. 1e), confirming the reversibility of the AID2 system.

### The AID2 system gives better control of degradation in mouse hippocampal neurons

**Fig. 2:**
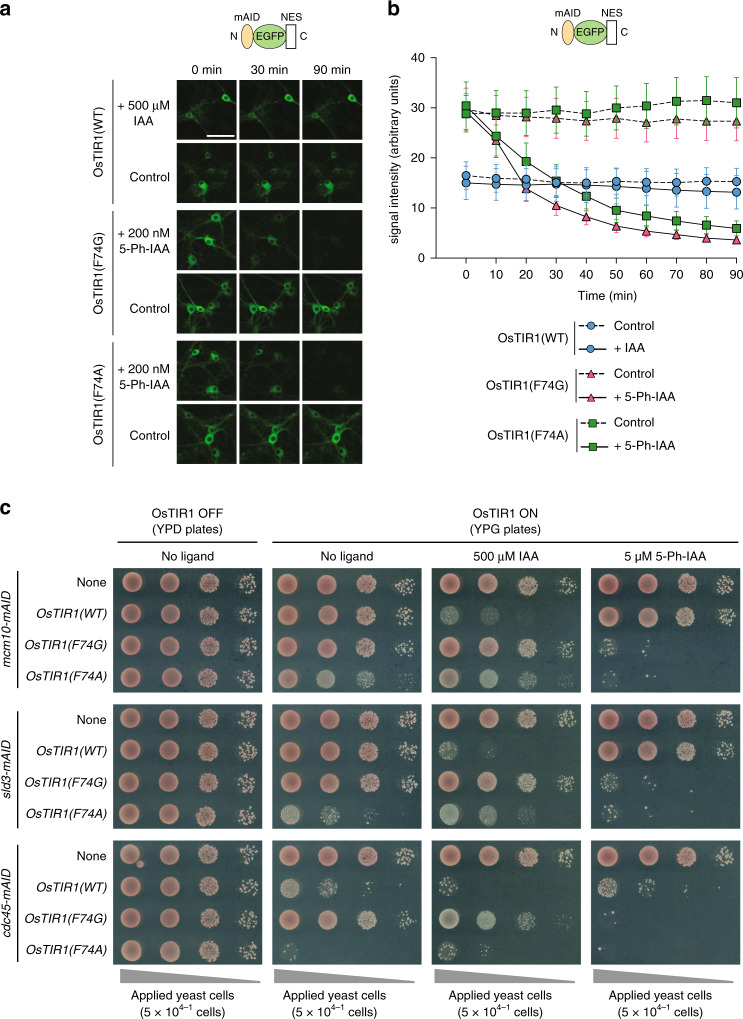
The AID2 systems work in mouse neurons and yeast. **a** Mouse hippocampal neurons expressing an mAID-EGFP-NES reporter together with OsTIR1(WT, F74G or F74A) were treated with the indicated ligand and time-course images were taken. We repeated each experiment at least twice and obtained similar results. Scale bar shows 50 µm. **b** A graph was drawn using the quantified EGFP signal from 30 cells. Data are presented as mean values of 95% confidence interval (*n* = 30 cells examined). **c** Haploid yeast lines having the indicated genotype were spotted on YPD (OsTIR1 OFF), YPG (OsTIR1 ON) and YPG containing 500 µM IAA or 5 µM 5-Ph-IAA. The plates were incubated at 25 °C for 2 (YPD plates) or 3 days (YPG plates).

To test AID2 system in another cell type, we chose mouse neurons derived from the hippocampus. We previously showed rapid degradation of another reporter, mAID-EGFP containing a nuclear export signal (mAID-EGFP-NES), in hippocampal neurons using OsTIR1(WT)^25^. We followed the same strategy of infecting cells with adeno-associated virus (AAV) harbouring OsTIR1(WT, F74G or F74A) and the mAID-EGFP-NES reporter (Supplementary Fig. 4A). Note that we infected with the viruses at approximately one-tenth of the titres previously used (4.7 × 10^9^ vg/mL for this experiment compared to 4.8 × 10^10^ vg/mL in the previous study)^25^. We quantified the expression level of OsTIR1 after immunostaining and did not see a significant difference between OsTIR1(WT), OsTIR1(F74G) and OsTIR1(F74A), possibly because the level of these proteins was high after viral infection (Supplementary Fig. 4B). The initial level of reporter expression was low in cells with OsTIR1(WT), which was suppressed by a proteasome inhibitor MG132, showing basal degradation by OsTIR1(WT) (Supplementary Fig. 4C). In cells with OsTIR1(WT), we did not detect IAA-dependent degradation under this condition (Fig. 2a, b). In sharp contrast, the reporter level was higher in cells expressing OsTIR1(F74G or F74A) and degradation was rapidly induced by the addition of 200 nM 5-Ph-IAA. The T1/2 was 17.5 and 24.8 min for OsTIR1(F74G) and OsTIR1(F74A), respectively (Fig. 2a, b). However, the two conditions did not differ significantly at any time point except for at 20 min (two-way ANOVA). These results supported the observations with human HCT116 cells and clearly showed that the AID2 system work not only in growing cells, but also in neurons that are terminally differentiated.

### The AID2 system allows generation of stricter yeast mutants

We previously showed that the original AID system worked in budding yeast^12^. To test whether AID2 also work in yeast and generate stricter mutants, we introduced the *OsTIR1(WT, F74G or F74A)* gene at the *URA3* locus under control of a *GAL1-10* promoter (Supplementary Fig. 4D). We subsequently tagged genes encoding an essential replication initiator, *MCM10*, *SLD3* or *CDC45*, with mAID. We showed previously that it was possible to generate a strict *cdc45* mutant^12^ but that it was difficult to generate strict *mcm10* and *sld3* mutants using OsTIR1(WT) combined with a single copy of mAID^13,26^. The mutant yeasts were tested on plates that suppressed or induced OsTIR1 expression (Fig. 2c). We found that the mutant strains expressing OsTIR1(WT or F74A) showed slow growth (Fig. 2c, OsTIR1 ON, no ligand, e.g., *cdc45-mAID*), suggesting that OsTIR1(WT) and OsTIR1(F74A) might have activated by IAA-like compounds within culture medium. OsTIR1(F74A) appeared to be more active under this condition. Moreover, OsTIR1(WT) and OsTIR1(F74A) could be activated by 500 µM IAA, although growth suppression was not complete with *mcm10-* or *sld3-mAID* strains expressing OsTIR1(WT)^13,26^. This is consistent with Supplementary Fig. 1E and the notion that AtTIR1(F79A) was still reactive to IAA^24^. In all cases, strains expressing OsTIR1(F74G or F74A) showed a profound growth defect on plates containing 5 µM 5-Ph-IAA (Fig. 2c and Supplementary Fig. 4E) and OsTIR1(F74A) worked slightly better at 1 µM 5-Ph-IAA (Supplementary Fig. 4F). Taken together, we concluded that the AID2 system can generate yeast mutants that show a stricter phenotype. Because OsTIR1(F74A) was more reactive to IAA than OsTIR1(F74G) in both yeast and human cells (Supplementary Fig. 1E and Fig. 2c), we focused on OsTIR1(F74G) in the rest of the following experiments.

### Sharper and quicker degradation of RAD21 cohesin

**Fig. 3:**
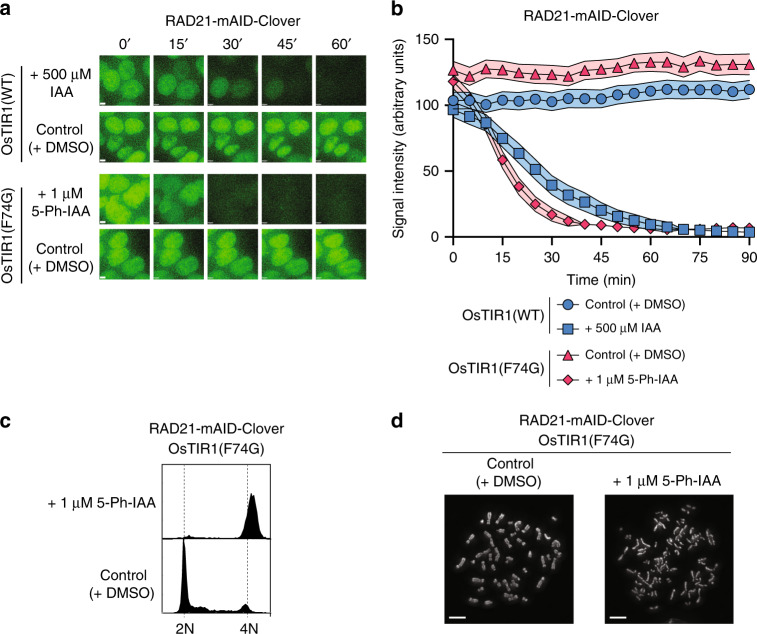
Comparison of endogenous RAD21 depletion by the AID and AID2 systems. **a** RAD21-mAC cells expressing OsTIR1(WT or F74G) were treated with the indicated ligand and time-course images were taken. We repeated each experiment at least twice and obtained similar results. Scale bars show 3.2 µm. **b** A graph was drawn using the quantified nuclear Clover signal from 60 cells. Data are presented as mean values of 95% confidence interval (*n* = 60 cells examined). **c** RAD21-mAC cells expressing OsTIR1(F74G) were treated with 1 µM 5-Ph-IAA for 24 h before flow cytometric analysis. **d** Mitotic chromosomes were assessed by a spread after treating RAD21-mAC cells expressing OsTIR1(F74G) with 1 µM 5-Ph-IAA for 2 h. We repeated this twice and obtained consistent results. Scale bar shows 5.4 µm.

Next, we wished to demonstrate control of a functional protein in human cells by the AID2 system employing the OsTIR1(F74G)–5-Ph-IAA pair. We previously showed that the endogenous RAD21 cohesin subunit fused with mAID-Clover could be rapidly targeted for degradation using the original AID system^15^. We generated similar RAD21-mAID-Clover (RAD21-mAC) cells expressing OsTIR1(F74G) by tagging endogenous RAD21 using CRISPR–Cas9 in the HCT116 background (Supplementary Fig. 5A). We noted that the level of RAD21-mAC was higher in cells expressing OsTIR1(F74G) than in cells expressing OsTIR1(WT) at zero time, again indicating the basal degradation was suppressed in those expressing OsTIR1(F74G) (Fig. 3b and Supplementary Fig. 5B). RAD21-mAC disappeared rapidly after the addition of the indicated ligand in both cases. However, the T1/2 for OsTIR1(WT) and OsTIR1(F74G) was 26.5 and 11.7 min, respectively, indicating that RAD21-mAC was degraded more quickly by the AID2 system, consistent with the case in Fig. 1d. These results indicate that the AID2 system allowed sharper and quicker control of RAD21-mAC than the original AID system.

To see the phenotype resulting after rapid RAD21-mAC depletion by the AID2 system, we looked at the cell cycle and found that the majority of RAD21-mAC-depleted cells were arrested in G2 or M phase (Fig. 3c). RAD21-mAC depleted cells showed a profound defect in sister chromatid cohesion, consistent with the essential role of this protein^27^ (Fig. 3d).

### The AID2 system allows generation of degron mutants that were difficult to establish using the AID system

**Fig. 4:**
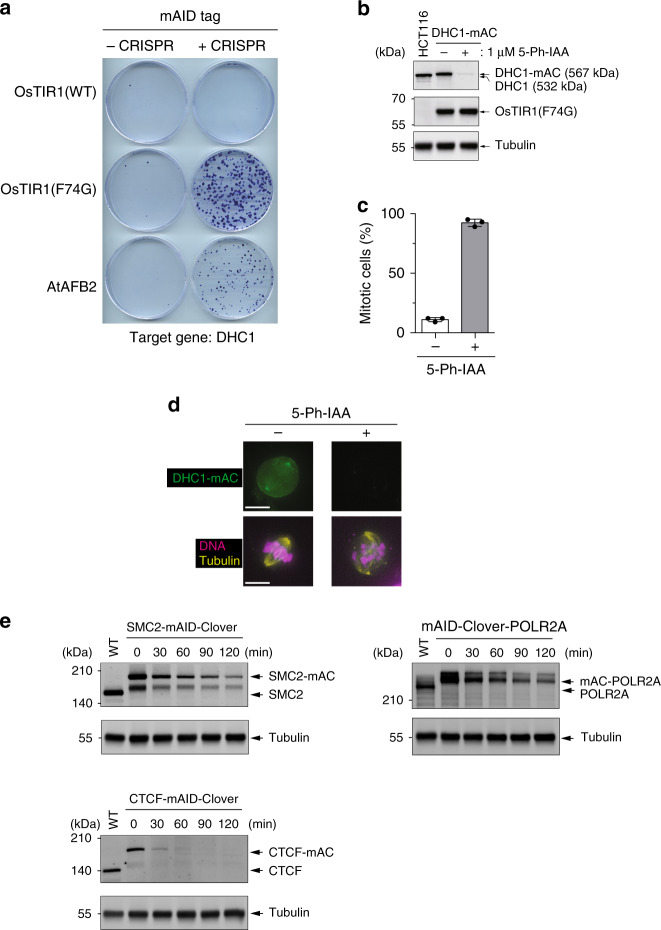
The AID2 system can generate conditional mutant cell lines for genes that were not possible to modify with the original AID system. **a** Colony formation of HCT116 cells expressing OsTIR1(WT), OsTIR1(F74G) or AtAFB2 after transfecting DHC1-tagging donors and a targeting CRISPR plasmid. Colonies were formed in the presence of 700 µg/ml of neomycin and 100 µg/ml of hygromycin for 11 days. **b** DHC1-mAC was induced to degrade in HCT116 cells expressing OsTIR1(F74G) by the addition of 1 µM 5-Ph-IAA for 6 h. DHC1-mAC and OsTIR1(F74G) were separated and detected by anti-DHC1 and -OsTIR1antibodies, respectively. Tubulin was used as a loading control. We repeated this experiment three times and obtained consistent results. **c** The mitotic index after DHC1-mAC depletion for 24 h. Data are presented as mean values ± SD (*n* = 3 independent experiments). **d** Microscopic image of mitotic cells after DHC1-mAC depletion. DHC1-mAC, DNA and tubulin were visualized. We repeated this experiment twice and obtained consistent results. Scale bars show 11 µm. **e** Indicated genes were tagged with mAID-Clover in HCT116 cells expressing OsTIR1(F74G). They were treated with 1 µM 5-Ph-IAA to take a time-course sample at the indicated time points. Proteins were separated and detected using a specific antibody. Tubulin was used as a loading control. We repeated this experiment three times and obtained similar results.

We previously reported that we could not generate a degron mutant for the cytoplasmic dynein heavy chain protein (DHC1) in HCT116 cells constitutively expressing OsTIR1(WT)^15^. This was because the basal degradation lowered the DHC1-mAID-Clover (DHC1-mAC) level, so that it was inadequate for cell survival. Using the fact that OsTIR1(F74G) showed a neglectable level of basal degradation (Fig. 1b), we wished to generate DHC1-mAC in cells constitutively expressing OsTIR1(F74G). The parental cell line constitutively expressing OsTIR1(WT or F74G) was transfected with a CRISPR plasmid for tagging and two donors harbouring a neomycin- or hygromycin-resistant marker, respectively^15^ (Supplementary Fig. 5A). Colonies were formed in the presence of G418 and hygromycin for DHC1 tagging at both alleles. No colonies were formed by the cells expressing OsTIR1(WT), consistent with our previous findings^15^ (Fig. 4a). In contrast, many colonies were formed by the cells expressing OsTIR1(F74G). In a clone expressing OsTIR1(F74G), DHC1-mAC was depleted efficiently by the addition of 1 µM 5-Ph-IAA (Fig. 4b). These cells were arrested in mitosis (Fig. 4c) and showed a strong defect in mitotic spindle formation (Fig. 4d), consistent with the essential function of DHC1^28,29^.

Our group and Ikonen’s group previously reported that AtAFB2, a TIR1 paralog in *Arabidopsis thaliana*, shows less basal degradation and can overcome the basal degradation-related inability to make degron mutants^21,30^. We succeeded in generating a DHC1-mAC mutant on the AtAFB2 background as reported by Li et al., although fewer colonies were obtained than when using the OsTIR1(F74G) background (Fig. 4a). Ikonen’s group also reported that another degron tag, mini-IAA7 (mIAA7), showed less basal degradation^21^ (Supplementary Fig. 6A). We also tested the mIAA7 tag fused with DHC1 in HCT116 cells expressing OsTIR1(WT), OsTIR1(F74G) or AtAFB2 (Supplementary Fig. 6B). In all cases, we successfully obtained colonies, consistent with the report that mIAA7 induces less basal degradation. However, compared with the OsTIR1(F74G)-expressing cells, fewer colonies were obtained with cells expressing OsTIR1(WT) or AtAFB2. We induced degradation of mIAA7- or mAID-tagged DHC1 in cells expressing AtAFB2 or OsTIR1(F74G) (Supplementary Fig. 6C). We found that the combination of OsTIR1(F74G) and mAID was the most efficient, supporting the idea that mIAA7 shows less basal degradation, but works less efficiently than mAID. This is possibly because the ternary complex formation by IAA is less efficient because of the shortness of mIAA7 and/or because mIAA7 has fewer lysine residues for ubiquitylation (Supplementary Fig. 6A).

Recently, the AID-ARF system composed of OsTIR1(WT), an AID/IAA17 tag and ARF16-BP1 (that binds the domains III and IV of AID/IAA17) suppresses the basal degradation of AID-fused proteins^23^ (Supplementary Fig. 6A). We established a parental HCT116 cell line expressing both OsTIR1(WT) and ARF16-BP1 and similarly tagged DHC1 with the AID/IAA17 tag (Supplementary Fig. 6D). We found that the cells expressing both OsTIR1(WT) and ARF16-BP1 did not form colonies. On the other hand, those expressing OsTIR1(F74G) formed many colonies. To confirm this observation, we generated cells expressing DHC1-AID/IAA17 in the original HCT116 background and subsequently introduced a donor vector for co-expression of OsTIR1(WT) and ARF16-BP1 at the AAVS1 locus. However, we failed to establish such cell lines. From these results, we concluded that DHC1-AID/IAA17 cells cannot be generated using the AID-ARF system, possibly because of incomplete suppression of the basal degradation.

We previously noted that it was not possible to generate an AID mutant of the SMC2 condensin subunit, CTCF insulator or RNA polymerase 2 largest subunit (POLR2A) using HCT116 cells constitutively expressing OsTIR1(WT), because their basal degradation killed the mutants^31,32^. Importantly, we successfully tagged both alleles of the endogenous SMC2, CTCF and POLR2A in HCT116 constitutively expressing OsTIR1(F74G) (Fig. 4e). In these cell lines, the mAID-fused targets were rapidly degraded by the addition of 1 µM 5-Ph-IAA. These results clearly showed that the AID2 system using cells constitutively expressing OsTIR1(F74G) can generate conditional mutants even for genes that were impossible to modify using the original AID system.

### The AID2 system can control protein expression in xenograft tumour in mice

Next, we wished to apply the AID2 system to control protein expression in living mice. For this purpose, we tested the suppression of xenograft tumour growth in nude mice. We initially tested the toxicity of 5-Ph-IAA by intraperitoneally injecting 0, 1, 3 or 10 mg/kg of 5-Ph-IAA every day for 1 week and observed no changes in body weight (Supplementary Fig. 7A).

**Fig. 5:**
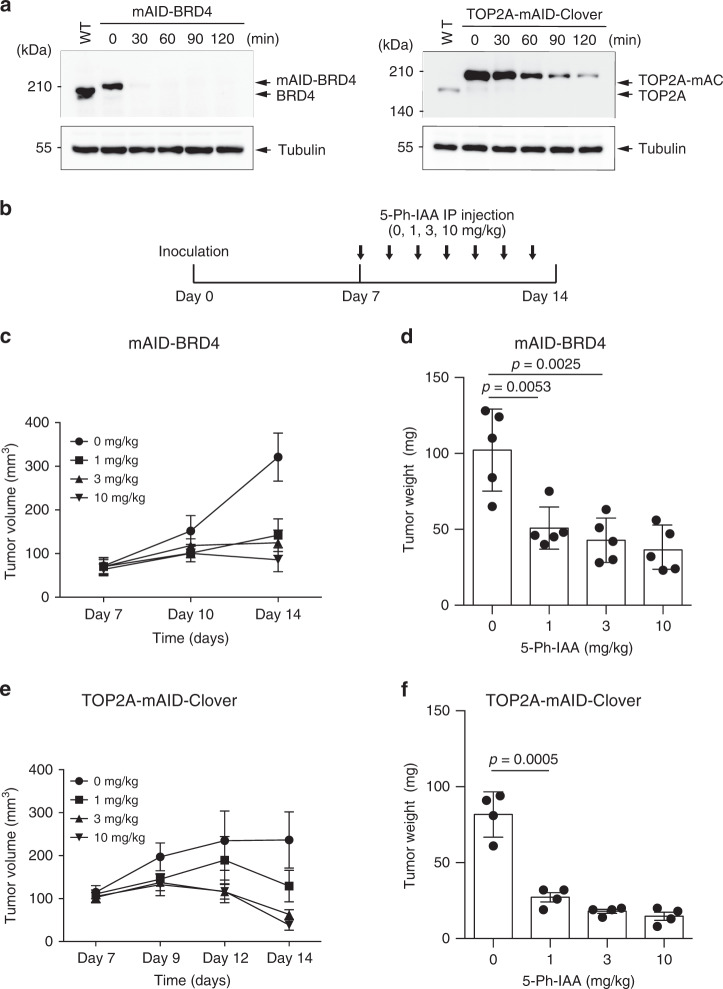
Xenograft tumour suppression by the AID2 system. **a** mAID-BRD4 or TOP2-mAC HCT116 cells expressing OsTIR1(F74G) were treated with 1 µM 5-Ph-IAA and sampled at the indicated time points. Proteins were separated and detected using a specific antibody. Tubulin was used as a loading control. We repeated this experiment twice and obtained similar results. **b** Experimental time-course diagram showing the xenograft assay. **c** Graph showing mAID-BRD4 xenograft tumour growth. Tumour volume was checked on the indicated days. Data are presented as mean values ± SD (*n* = 5 animals). **d** Graph showing the tumour weight on day 14. mAID-BRD4 xenograft tumour was taken and weighed. Data are presented as mean values ± SD (*n* = 5 animals, two-tailed *t*-test). **e** Graph showing TOP2A-mAC xenograft tumour growth. Tumour volume was checked on the indicated days. Data are presented as mean values ± SD (*n* = 4 animals). **f** Graph showing the tumour weight on day 14. TOP2A-mAC xenograft tumour was taken and weighed. Data are presented as mean values ± SD (*n* = 4 animals, two-tailed *t*-test).

Inhibitors of BRD4 and TOP2A are potential anticancer drugs^33,34^. To model cancer suppression by these drugs, we generated BRD4- and TOP2A-degron cell lines using the HCT116 CMV-OsTIR1(F74G) background and confirmed the rapid degradation of the fusion proteins (Fig. 5a). Note that the expression level of TOP2A-mAC (TOP2A-mAID-Clover) is enhanced compared to that of untagged TOP2A, possibly because the insertion of the selection marker might have destroyed a 3′ UTR that affects the expression control or the C-terminal tagging with mAID-Clover might have stabilized the fusion protein. We transplanted mAID-BRD4 or TOP2A-mAC cells under mouse skin and xenograft tumours formed over 1 week. We subsequently administered 0, 1, 3 or 10 mg/kg of 5-Ph-IAA by intraperitoneal (IP) injection every day for another week (Fig. 5b). In a control experiment in which we xenografted the parental HCT116 cells expressing OsTIR1(F74G), no tumour suppression was seen (Supplementary Fig. 7B–D). In contrast, we observed significant tumour suppression of mAID-BRD4 xenografts at all doses of 5-Ph-IAA treatment (Fig. 5c, d and Supplementary Fig. 7D). A similar trend was seen when we used TOP2A-mAC xenografts (Fig. 5e, f). These results suggest that, using the AID2 system, mAID-BRD4 and TOP2A-mAC could be successfully depleted in living mice, causing tumour suppression.

For comparison, we generated similar TOP2-mAC cells using the original AID system (Supplementary Fig. 8A). We similarly transplanted this cell line to monitor tumour growth after administrating 0, 10, 30, 100 mg/kg of IAA, which are ten times higher doses than those used in the above xenografts with 5-Ph-IAA (Supplementary Fig. 8B–D). We did not observe tumour suppression in an IAA-dependent manner. Taken together, we concluded that AID2, but not AID, works in tumour xenograft assays using mice.

### The AID2 system works rapidly in multiple organs in mice

**Fig. 6:**
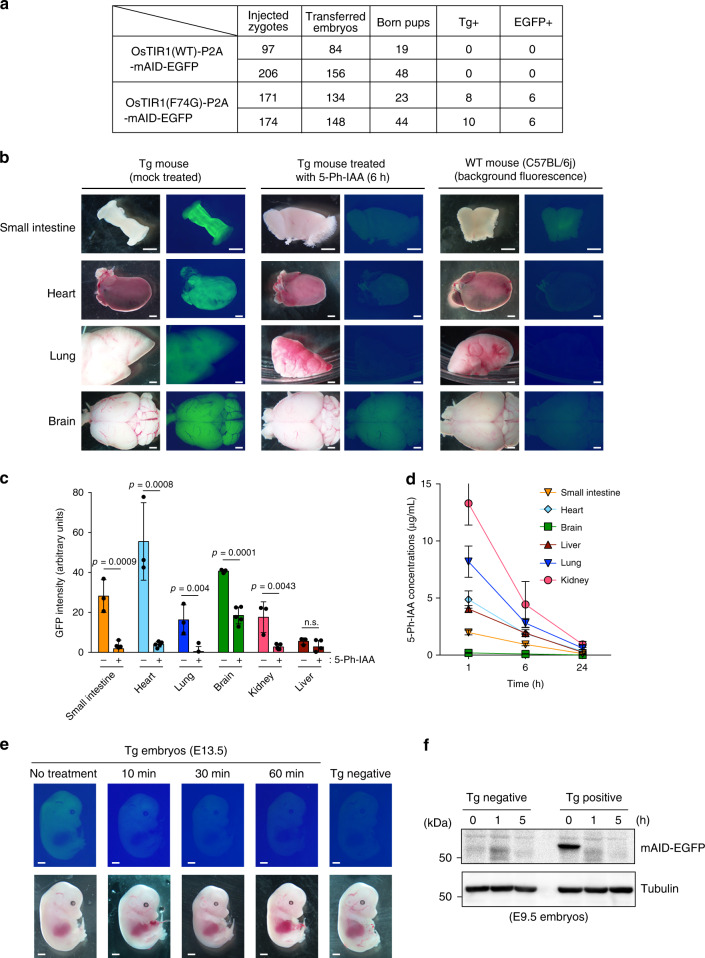
Rapid depletion of an mAID-EGFP reporter in living TG mice. **a** A table showing the results of microinjection using two different transgenes. The number of zygotes, transferred embryos, pups born, PCR-positive pups and EGFP-positive pups obtained from the two independent experiments for each transgene were shown. **b** Two female mice derived from the Tg #1 line were mock treated or administered 5 mg/kg of 5-Ph-IAA. Indicated organs were removed after 6 h and photographed using the same exposure condition. Scale bars show 1 mm. **c** GFP intensity of the data shown in (**b**) and Supplementary Fig. 9B was quantified and subtracted from the average intensity of WT. Data are presented as mean values ± SD (*n* = 3 and 5 animals for control and 5-Ph-IAA, respectively, two-tailed *t*-test). **d** Tissue distribution of 5-Ph-IAA after injection of 5 mg/kg of 5-Ph-IAA. Data are presented as mean values ± SD (*n* = 3 animals). **e** Pregnant wild-type female mice crossed with a male of the Tg #2 line were administered 5 mg/kg of 5-Ph-IAA at the E13.5 stage and the mothers were sacrificed at the indicated time points to examine the Tg-positive embryos. Scale bars show 1 mm. **f** Pregnant wild-type female mice crossed with a male of the Tg #2 line were administered 5 mg/kg of 5-Ph-IAA at the E9.5 stage. Subsequently, protein extracts of the embryos were prepared at the indicated time points. The reporter was separated and detected using anti-mAID antibody. Tubulin was used as a loading control. We repeated this experiment three times and obtained similar results.

To further test whether the AID2 system works in living mice, we wished to establish transgenic (Tg) mice expressing OsTIR1(WT or F74G) together with an mAID-EGFP reporter. To our surprise, we failed to establish mouse lines expressing OsTIR1(WT), suggesting that OsTIR1(WT) causes an unknown lethal problem during embryogenesis (Fig. 6a). In sharp contrast, we successfully established multiple mouse lines expressing OsTIR1(F74G) together with the reporter. Because of the nature of random integration of the transgene, each line showed different expression patterns. We chose two Tg lines (#1 and #2), that showed a stronger level of the expression in multiple organs, for further analysis.

Adult mice derived from the Tg line #1 were used to test the reporter depletion in organs. Three mice (one male and two females) were mock treated and five mice (two males and three females) were treated with 5 mg/kg of 5-Ph-IAA by IP injection. After 6 h, the reporter expression in organs was examined. Compared with the control, reporter expression was significantly reduced to a background level in multiple organs including the small intestine, heart, lung and kidney (Fig. 6b, c and Supplementary Fig. 9A). We observed that, in the brain, reporter expression was reduced but was not complete (Fig. 6b, c, brain), possibly because the distribution of 5-Ph-IAA to the brain was not efficient (Fig. 6d). Another possible reason was that the level of the E3 SCF–OsTIR1(F74G) ligase was not high enough in the brain, although the depletion took place efficiently in neurons grown on a plate (Fig. 2a). These results clearly indicate that the AID2 system can control protein expression in multiple organs in vivo.

We also tested whether this system can be applied for embryogenic stage by injecting 5-Ph-IAA into pregnant females crossed with the Tg line #2. Pregnant females with E13.5 embryos were treated with 5 mg/kg of 5-Ph-IAA by IP injection. Subsequently, embryos were recovered after 10, 30 and 60 min after injection. The reporter signals quickly disappeared within 60 min (Fig. 6e and Supplementary Fig. 9B). We also confirmed rapid depletion of the reporter in E9.5 embryos (Fig. 6f and Supplementary Fig. 9C). These results indicate the rapid delivery of 5-Ph-IAA from the mother to embryos and depletion of the reporter in embryos.

## Discussion

We developed the AID2 system by employing a bump-and-hole strategy and here describe its advantages over the original AID system. An important feature of the AID2 system is the use of a ligand, 5-Ph-IAA, which works efficiently at <1 µM (Figs. 1–4), a concentration that did not cause any significant defects in human cell cultures (Supplementary Fig. 3). Why does 5-Ph-IAA work at much lower concentrations than IAA? We found that OsTIR1(F74G) is more stable than OsTIR1(WT), possibly because self-degradation was suppressed by the F74G mutation (Supplementary Fig. 1D). This must contribute to the efficient activation of OsTIR1(F74G) at lower concentrations of 5-Ph-IAA. In addition, as previously reported by Uchida et al.^24^, the formation of ternary complexes composed of OsTIR1(F74G)/5-Ph-IAA/mAID is likely to be more efficient than that involving IAA. We also speculate that 5-Ph-IAA might be more cell permeable and metabolically stable than IAA. Indeed, 5-Ph-IAA and IAA have logP of 2.8 and 1.1, respectively, showing that 5-Ph-IAA is more lipophilic and thus is likely more cell permeable. A combination of these features might explain why 5-Ph-IAA works at significantly lower concentrations than IAA.

Another important feature of the AID2 system is that the basal degradation in the absence of ligand is neglectable (Figs. 1b–d, 2 and 3a, b). The main source of basal degradation is considered to be indole chemicals, including IAA, that are present in culture medium (mainly derived from bovine serum). We found that OsTIR1(F74G) is less reactive to IAA (Supplementary Fig. 1E and Fig. 2c), thereby contributing to suppression of basal degradation. It has been reported that AtTIR1 has a weak affinity to IAA7 even in the absence of IAA^35^, suggesting that mAID might have a weak affinity for OsTIR1(WT) without IAA. We speculate that OsTIR1(F74G) might have a lower affinity for mAID in the absence of its activating ligand.

To compare AID2 with the other improved AID systems reported to show lower leaky degradation^21,23^, we summarized the features and differences among these systems in Supplementary Fig. 10. By taking DHC1 as an example to evaluate the level of basal degradation, we showed that DHC1-degron cells could be established by using the AtAFB2-mIAA7 system (Supplementary Fig. S6B). However, in these cells, DHC1 depletion was less efficient than in DHC1-mAID cells established using AID2 (Supplementary Fig. 6C). In the case of the ARF-AID system, we could not establish DHC1-AID/IAA17 cells (Supplementary Fig. 6D). Taking the fact that AID2 works with lower ligand concentrations, these results suggest that AID2 is the best option for avoiding basal degradation and achieving sharp target depletion.

Using the OsTIR1(F74G)–5-Ph-IAA pair, we showed that in mammalian cells: (1) the DC50 value is about 670 times lower (Fig. 1c), (2) the basal degradation is undetectable (Fig. 1b) and (3) the depletion speed is faster (Figs. 1d and 3b) than in the original AID system. A combination of these advantages allows us to generate conditional human mutant cells for genes that were previously difficult to deal with^15^, and to rapidly deplete these target proteins with a very low concentration of 5-Ph-IAA (Fig. 4). This, combined with its reversibility (Fig. 1e), means that the AID2 system using the OsTIR1(F74G)–5-Ph-IAA pair will in future contribute to broad areas of cell biology. Moreover, we describe further advantages of the AID2 system: (4) stricter mutants can be generated in yeast (Fig. 2c) and (5) rapid depletion can be achieved in living mice (Figs. 5 and 6). The use of the original AID system has been limited in mouse zygotes and early embryos^36,37^, possibly because mouse lines constitutively expressing OsTIR1(WT) cannot be generated (Fig. 6a). The AID2 system with the OsTIR1(F74G)–5-Ph-IAA pair overcomes this critical problem and opens doors not only to yeast and cell genetics, but also to mouse genetics.

We noted that depletion of the reporter in the mouse brain was not as efficient as in other organs (Fig. 6b, c). Brain distribution of 5-Ph-IAA was not efficient (Fig. 6d), possibly because the blood–brain barrier blocks 5-Ph-IAA. A modified 5-Ph-IAA analogue or another reported ligand, 5-adamantyl-IAA^38^, might show better brain distribution. It will be of interest to test the usefulness of these ligands for controlling protein expression in the brain.

In this paper, we described the rapid depletion of nuclear and cytoplasmic proteins in proliferating and non-proliferating mammalian cells using the AID2 system with the OsTIR1(F74G)–5-Ph-IAA pair (Figs. 1–4). We and others showed that membrane proteins can be depleted using the original AID system^12,21^, so that the control of membrane proteins should be more efficient using AID2. The original AID system has already been applied to many uni- and multi-cellular organisms including fission yeast^39^, the parasite *Toxoplasma gondii*^40^, *Drosophila*^41,42^, *C. elegans*^43^ and zebrafish^44^. We predict that AID2 system can also be applied to these organisms.

## Methods

### Plasmids

All plasmids used in this study are listed in Supplementary Table 2. These plasmids and their sequence information are available from Addgene and RIKEN-NBRP.

### Chemical synthesis of ligands

Ligands including 5-Ph-IAA were synthesized as described in Supplementary Methods. 5-Ph-IAA is commercially available as a reagent (BioAcademia, Japan, #30-003).

### Cell culture

All HCT116 cell lines used in this study are listed in Supplementary Table 3. HCT116 cells (ATCC, #CCL-247) were cultured in McCoy’s 5A, supplemented with 10% FBS (Gibco, #26140-079), 2 mM L-glutamine, 100 U/ml penicillin and 100 μg/ml streptomycin at 37 °C in 5% CO_2_. Cells were transfected with CRISPR–Cas9 and donor plasmids using FuGENE HD Transfection Reagent (Promega, #E2311) in a 12-well plate. One day after transfection, cells were plated in 10 cm dishes and selected with antibiotics. Selected clones were isolated and confirmed by following a published protocol^22^. IAA, 5-(3-MeOPh)-IAA, 5-Ph-IAA, 5-(3,4-dimethylphenyl)-indole-3-acetic acid, 5-(3-methylphenyl)-indole-3-acetic acid and 5-(3-chlorophenyl)-indole-3-acetic acid were dissolved in DMSO to make a 500 mM stock solution, and further diluted with DMSO to an appropriate concentration before the experiment. In experiments using cells expressing a reporter (mAID-EGFP-NLS/NES), the culture medium was replaced with the medium containing an appropriate concentration of ligands. For inducing degradation of an endogenous protein fused with mAID, IAA or 5-Ph-IAA was added directly to the culture medium at an appropriate concentration.

### AAV transfection and culture of mouse neurons

AAV vector was generated as described previously^25,45^. HEK293T cells were transfected with pAAV-hSyn-OsTIR1(WT, F74G or F74A) together with two AAV helper plasmids encoding serotype DJ (Cell Biolabs, #VPK-400-DJ) using polyethylenimine (Polysciences, #24765-1). Three days after transfection, AAVs were collected from the transfected cells and purified using AAVpro Purification Kit (Takara Bio, #6666) according to the manufacturer’s protocol. The viral titre was determined by real-time PCR using ITR2 primers^46^.

Dissociated hippocampal neurons were prepared from a postnatal C57BL/6J mouse (day 0) as previously described^47^. Hippocampal tissue was dissected and minced in prewarmed Hank’s balanced salt solution (HBSS; Sigma-Aldrich, #H9269) and treated with 0.25% Trypsin/EDTA at 37 °C. After 15 min of incubation, the tissue was treated with DNaseI (Sigma-Aldrich, #10104159001) at room temperature for 5 min and subsequently washed with HBSS three times. HBSS was replaced with neurobasal plating medium [Neurobasal Medium containing B27 Supplement (Gibco, #17504-044) (1:50), 0.5 mM Glutamine Solution, 25 μM Glutamate, Penicillin/Streptomycin (1:200), 1 mM HEPES, 10% horse serum (Gibco, #26050-088)]. Tissue was triturated using fire-polished Pasteur pipettes and filtered through a 40-μm cell strainer (Corning, #352340). Hippocampal cells were plated on a 35 mm glass-bottom dish (12 mm diameter × 0.15 mm thickness glass; Iwaki, #11-0602), coated with poly-d-Lysine at a density of 4.0 × 10^4^ cells/well and cultured in a 37 °C incubator with 5% CO_2_. On day 1 in vitro culture (DIV), neurobasal plating medium was replaced with serum-free neurobasal feeding medium [neurobasal medium containing B27 supplement (1:50), 0.5 mM Glutamine Solution, Penicillin/Streptomycin (1:200), 1 mM HEPES]. On DIV2, the medium was replaced with fresh feeding medium containing 5 μM cytosine β-d-arabinofuranoside (AraC; Sigma-Aldrich, #C-1768) for 24 h to inhibit the growth of non-neuronal cells. The medium was replaced with fresh feeding medium 24 h after the AraC addition. After DIV3, half of the neurobasal medium was replaced with a fresh neurobasal feeding medium every 4 days. 1 μl of the diluted AAV (4.8 × 10^12^ vg/ml) was dropped into the culture on DIV7 and the degradation assay was performed on DIV14 or 15.

Images were acquired using an FV1200 scanning confocal microscope (Olympus) equipped with diode lasers. For imaging primary culture, Z-series images (five optical sections) were acquired with a ×10 water immersion objective lens (0.40 numerical aperture, Olympus). EGFP signal intensities within soma were measured using an ImageJ software. To quantify the expression level of OsTIR1, neurons on DIV14 were immuno-stained with anti-OsTIR1 (MBL, #PD048) and then with Alexa Fluor 555 anti-rabbit IgG (ThermoFisher, #A-31572).

### Yeast culture

In this study, W303-1a haploid yeast was used. To generate yeast lines expressing OsTIR1(WT, F74G or F74A), W303-1a was transfected with linearized pMK198, pMK419 or pMK425 by StuI, respectively. After selection on a SD-Ura plate, clones were confirmed by genomic PCR and western blotting to detect OsTIR1 in the presence of galactose. Confirmed clones were stocked as parental lines.

To fuse the mAID tag at the C-terminal coding locus of the *MCM10*^26^, *SLD3*^13^ and *CDC45*^12^ genes, a DNA fragment composed of mAID and a hygromycin-resistant marker was PCR amplified from pSM412 by using the oligonucleotides following the published protocol^48^.

*MCM10*: 5′-AGGAAACTAAAGAAACTTCTGACGGTAGTGCCAGCGATCTTGAGATAATACGTACGCTGCAGGTCGAC-3′; 5′-TTCTTTCTCACTTTAAGGATTGATTCCCTATATTGCAACCAAAATCACTCATCGATGAATTCGAGCTCG-3′

*SLD3*: 5′-ATAGCTCAAAAAGGAGAGTAAGAAGACGTTTATTTGCTCCAGAATCCACACGTACGCTGCAGGTCGAC-3′; 5′-TTTAATTGTATACTCAAAGGCCCCCGAAGTGCGAAATTGTTGTAGCTTAGATCGATGAATTCGAGCTCG-3′

*CDC45*: 5′-GTGAAGATCTTTCACCATTCCTGGAGAAGCTGACCTTGAGTGGATTGTTACGTACGCTGCAGGTCGAC-3′; 5′-GTTGGACTTAAAAGCTTGAAAAAGCTTAGATTTTATATTCATATGCTGGTATCGATGAATTCGAGCTCG-3′

Amplified DNA was used for transformation of the parental lines. Clones were selected on YPD plates containing 300 µg/ml of hygromycin B. The insertion was confirmed by genomic PCR and the expression of mAID-fused protein was checked by western blotting. To test growth phenotype, 5 × 10^4^, 5 × 10^3^, 5 × 10^2^ and 5 × 10^1^ cells were spotted on a YPD or YPG plate.

### Transcriptome analysis

HCT116 cell line and a line expressing OsTIR1(WT) or OsTIR1(F74G) were seeded on a six-well plate and treated with 100 µM IAA or 1 µM 5-Ph-IAA, respectively, for 24 h before RNA extraction using an illustra RNAspin Mini kit (GE healthcare, #25-0500-70). Libraries were prepared using TruSeq Stranded mRNA Library Prep kit (Illumina, #20020594) and subsequently sequenced using Illumina HiSeq2500 and HiSeq4000 to obtain more than 1.2 × 10^7^ reads. Raw sequencing data reported are available in the DNA Data Bank of Japan (DDBJ) Sequencing Read Archive under the accession numbers DRA009832 and DRA010661.

Data pre-processing and quality control of raw sequence reads were conducted by Trimmomatic^49^. After trimming the adaptor sequences, we removed the sequence reads having a base-quality score < 25. The low-quality bases (base quality < 20) at the head and tail of each read were trimmed. A sliding window trimming was implemented if the average quality within a four-base wide window were lower than 20. We excluded the sequences, the length of which was <50 after the trimming processes.

The pre-processed sequences were aligned to the human reference genome (GRCh37) by using a splice-aware RNA-seq alignment tool, GSNAP^50^. We retrieved the annotated gene model in gene transfer format (GTF) from the GENCODE (human release 28)^51^. We aligned the sequence reads to both known and newly identified splice junctions. The Sequence Alignment Map (SAM) files generated by the GSNAP aligner were converted to Binary Alignment Map files via SAMtools^52^. The counts of reads that uniquely mapped to exons were summarized at the gene level defined by the GTF file by using featureCounts^53^. The raw expression counts were converted to counts per million (CPM). After adding an offset of 1, the CPM values were further converted to the log2-scale. Fold changes of gene expression levels between ligand- and vehicle-treated cell lines were calculated for genes whose CPM values were >0.5 in at least one sample. We performed Fisher’s exact test for the genes showing a fold-change of at least 1.5 to assess the following two hypotheses for the advantage of AID2 system: (1) the impact of 1 µM 5-Ph-IAA treatment on gene expression profile is weaker than that of 100 µM IAA treatment, and (2) the impact of AID2 system (1 µM 5-Ph-IAA treatment in OsTIR1 (F74G) expressing cells) on gene expression profile is weaker than that of AID system (100 µM IAA treatment in OsTIR1 (WT) expressing cells). Statistical analysis was performed using the computing environment R (https://www.r-project.org/). Odds ratios, 95% confidence intervals and two-sided *P* values were estimated.

### Flow cytometric analysis

HCT116 cells were seeded at 1 × 10^5^ cells/well in a six-well plate and grown for 2 days. In case of inducing the expression of OsTIR1(WT or F74G) under the control of the Tet promoter (Supplementary Fig. 1F), cells were treated with 0.5 µg/mL of doxycycline for 24 h, and then 100 µM IAA or 1 µM 5-Ph-IAA was added. For detecting EGFP and Clover signals after ligand treatment, cells were trypsinized and fixed in 4% methanol-free paraformaldehyde phosphate buffer (FUJIFILM Wako Pure Chemical Corporation) at 4 °C overnight. Fixed cells were washed and resuspended in PBS containing 1% BSA. To detect OsTIR1-V5 in Supplementary Fig. 1F, fixed cells were treated with anti-V5 antibody (Invitrogen, #R960-25) and subsequently stained with Alexa Fluor 647 anti-mouse IgG (ThermoFisher, #A-21236). For measuring the DNA signal after ligand treatment, cells were trypsinized and fixed in 70% EtOH. Fixed cells were washed, resuspended in PBS containing 1% BSA, 50 μg/ml of RNase A, and 40 μg/ml of propidium iodide, and incubated at 37 °C for 30 min. Flow cytometric analysis was performed on a BD Accuri C6 machine (BD Biosciences) using FCS4 Express Cytometry software (DeNovo Software). Ten thousand cells were analyzed from each sample, except in the case of Supplementary Fig. 1F, in which 50,000 cells were analyzed.

### Protein detection

HCT116 cells were seeded at 1 × 10^5^ cells/well in a six-well plate and grown for 2 days. After 1 µM 5-Ph-IAA treatment, cells were lysed in RIPA buffer (25 mM Tris-HCl pH7.6, 150 mM NaCl, 1% NP40, 1% sodium deoxycholate, 0.1% SDS). After centrifugation, the supernatant was mixed with 2 × SDS sample buffer (Tris-HCl pH6.8, 4% SDS, 20% glycerol, 10% 2-mercaptoethanol, 0.004% bromophenol blue) before incubation at 95 °C for 5 min. Equal amounts of protein (measured using Bradford (Bio-Rad Smart Spec 3000) assay) were loaded onto a TGX Stain-Free gel (Bio-Rad) and transferred onto a Hybond ECL membrane (GE Healthcare). The membrane was incubated with a primary antibody at 4 °C overnight and subsequently incubated with a secondary antibody at room temperature for 3 h. Detection was performed using the Amersham ECL Prime reagents (GE Healthcare) in case of using an HRP-conjugated secondary antibody and images were acquired with a ChemiDoc Touch MP system (Bio-Rad).

A mouse embryo at the E9.5 stage was lysed in TNE buffer (50 mM Tris-HCl pH7.4, 150 mM NaCl, 1% NP40, 1 mM EDTA, 1 mM DTT) with cOmplete Protease Inhibitor cocktail (SIGMA-Aldrich, #4693132). The following steps were the same as above.

### Antibodies

For protein detection, the following commercially available antibodies were used. Primary antibodies: anti-OsTIR1 (MBL, #PD048), anti-mAID (MBL, #M214-3), anti-DHC1 (SantaCruz, #sc-9115), anti-SMC2 (Bethyl, #A300-058A-T), anti-CTCF (Bethyl, #A300-543-T), anti-POLR2A (Abcom, #ab817), anti-BRD4 (GeneTex, #GTX130586), anti-TOP2A (MBL, #M042-3S), anti-alpha-tubulin (MBL, #M175-3), anti-beta-tubulin (SIGMA-Aldrich, #T4026), anti-alpha-tubulin conjugated with rhodamine (Bio-Rad, 12004165). All primary antibodies were used at a 1 in 1000 dilution with TBST containing 5% skim milk. Secondary antibodies: anti-rabbit IgG HRP (GE Healthcare, #NA934), anti-mouse IgG HRP (SantaCruz, #PI-2000), anti-rabbit IgG StarBright Blue 700 (Bio-Rad, #12004161). All secondary antibodies were used at a 1 in 5000 dilution with TBST containing 5% skim milk.

### Microscopy

HCT116 cells cultured in McCoy’s 5A medium without phenol red, supplemented with 10% FBS (Gibco, #26140-079), 2 mM L-glutamine, 100 U/ml penicillin and 100 μg/ml streptomycin were imaged on a DeltaVision deconvolution microscope (GE Healthcare) equipped with an incubation chamber and a CO_2_ supply system. To visualize nuclei, 0.2 μM SiR-DNA (Spirochrome) was added for 3 h (Fig. 3a) or 24 h (Fig. 4d) before observation. To visualize tubulin, cells were treated with CellLight^TM^ Tubulin-RFP, BacMam 2.0 (ThermoFisher Scientific) for 24 h before observation (Fig. 4d). To calculate the mitotic index shown (Fig. 4c), brightfield images after 5-Ph-IAA treatment were acquired using an EVOS XL Core Configured Microscope (ThermoFisher Scientific).

### Chromosome spread

HCT116 cells were cultured to 70% confluency in a 60-mm dish. KaryoMAX^TM^ Colcemid^TM^ Solution in PBS (Gibco, #15212012) was added to a final concentration of 0.02 μg/ml together with DMSO (control) or 1 µM 5-Ph-IAA. Treated cells were incubated at 37 °C 5% CO_2_ for 2 h before trypsinization. Removed cells were treated with 75 mM KCl before fixation in MeOH/acetic acid (3:1) fixative solution. Fixed cells were adjusted to approximately 10^7^ cells/ml. 10 μl of the cell suspension was applied onto a glass slide and dried at room temperature. 10 μl of DAPI-containing Vectashield Mounting Medium (Vector Laboratories, #H-1200) was added before sealing with a coverslip. Chromosomes were observed under a DeltaVision deconvolution microscope (GE Healthcare).

### Animal experiments

Nude mice used for xenograft assay were Balb/c-nu female mice (7 weeks old) weighing 16–20 g and were obtained from Charles River Japan (Kanagawa, Japan). These animals were acclimated at least for 1 week before use. Indicated HCT116 lines (1 × 10^5^ cells for mAID-BRD4 and 2 × 10^5^ cells for TOP2A-mAC) were resuspended in 0.1 ml of HBSS (Sigma-Aldrich, #H9269) containing 0.05 ml of Matrigel (Corning, #356237). The suspension was injected into the both sides of flank. Six or 7 days after IAA or 5-Ph-IAA injection, respectively, the mice were randomized and treated daily with the indicated dose of IAA or 5-Ph-IAA by IP injection for additional 6 or 7 days. Tumour volume (=*L* × *W* × *W*/2; *L* and *W* stand for the longest and shortest diameters, respectively) was measured on the indicated days. At the end of the experiment, xenograft tumour was removed and weighed.

To generate Tg mice, B6C3F1 (C57BL/6N X C3H/HeN) female mice (4 or 5 weeks old) were super-ovulated and mated with B6C3F1 males (3–12 months old). Fertilized embryos were collected from oviducts. A vector plasmid (pMK427 or pMK411) was linearized with AflII and MluI before an appropriate DNA fragment was purified from an agarose gel. The purified DNA fragment was dissolved in injection buffer (10 mM Tris-HCl and 0.1 mM EDTA, pH7.5), and injected into the pronucleus of fertilized eggs in M2 media (Sigma-Aldrich, #M1767). The injected zygotes were cultured in KSOM media (Millipore, #MR-121-D) at 37 °C under 5% CO_2_ until the two-cell stage after 1.5 days. Thereafter, 20–32 embryos at the two-cell stage were transferred into the uterus of pseudo-pregnant MCH females at 0.5 dpc, and let them be born naturally. Tg mice were identified by PCR with GFP L1 (5′-CCTGGTCGAGCTGGACGGCGAC-3′) and GFP R1 (5′-TCACGAACTCCAGCAGGACCATG-3′) primers.

To treat mice with 5-Ph-IAA, 5-Ph-IAA dissolved in PBS was intraperitoneally injected. Images of tissues and embryos were taken with a LEICA MZ16F stereomicroscope equipped with an OLYMPUS DP74 camera. GFP intensity was measured by using the ImageJ software. Green channel images were saved as 8 bit grayscale images. The tissue or embryo outlines were selected for measuring the mean grey value. The mean grey value of a rectangle area nearby the object was also measured as a background, which was subtracted from the intensity value of the object.

All animals were kept in a room conditioned at 23 ± 2 °C, with 50 ± 10% humidity and under a 12 h light-and-dark cycle. All protocols and procedures involving the care and use of animals were reviewed and approved by the Institutional Animal Care and Use Committee of National Institute of Genetics and The University of Tokyo prior to conduct. Throughout the study, the care and use of animals were conducted in accordance with the guidelines and regulations set by the Ministry of Education, Culture, Sports, Science and Technology, the Ministry of the Environment and the Science Council of Japan.

### Data analysis

We used FSC Express 4, Volocity 6.3.1, ImageJ, SAMtools, Trimmomatic, GSNAP, featureCounts and R for data analysis. We used GraphPad Prism 6 for statistical analysis and making all graphs.

### Reporting summary

Further information on research design is available in the Nature Research Reporting Summary linked to this article.

## Supplementary information

Supplementary Information

Reporting Summary

Peer Review File

## Data Availability

The transcriptome data shown in Supplementary Fig. 3C are available in the DNA Data Bank of Japan (DDBJ) Sequencing Read Archive under the accession numbers DRA009832 and DRA010661. All plasmids listed in Supplementary Table 2 are available from Addgene and RIKEN-BRC. The cell lines are listed in Supplementary Table 3. Source data are provided with this paper.  All other data that support the findings of this study are available from the corresponding author upon request.

## Source data

Source Data
