# Supplementary material for: The auxin-inducible degron 2 technology provides sharp degradation control in yeast, mammalian cells, and mice
Source: Nat Commun. 2020 Nov 11;11:5701. doi: 10.1038/s41467-020-19532-z (PMC7659001; doi:10.1038/s41467-020-19532-z)
Supplement: Supplementary file 1 — Supplementary Information [file 41467_2020_19532_MOESM1_ESM.pdf]

The auxin-inducible degron 2 technology gives superior degradation control in yeast, mammalian cells, and mice

Aisha Yesbolatova, Yuichiro Saito, Naomi Kitamoto, Hatsune Makino-Itou, Rieko Ajima, Risako Nakano, Hirofumi Nakaoka, Kosuke Fukui, Kanae Gamo, Yusuke Tominari, Haruki Takeuchi, Yumiko Saga, Ken-ichiro Hayashi, and Masato T. Kanemaki

List of Supplementary Information

- **Supplementary Figures 1–10**
- **Supplementary Tables 1–3**
- **Supplementary Methods**

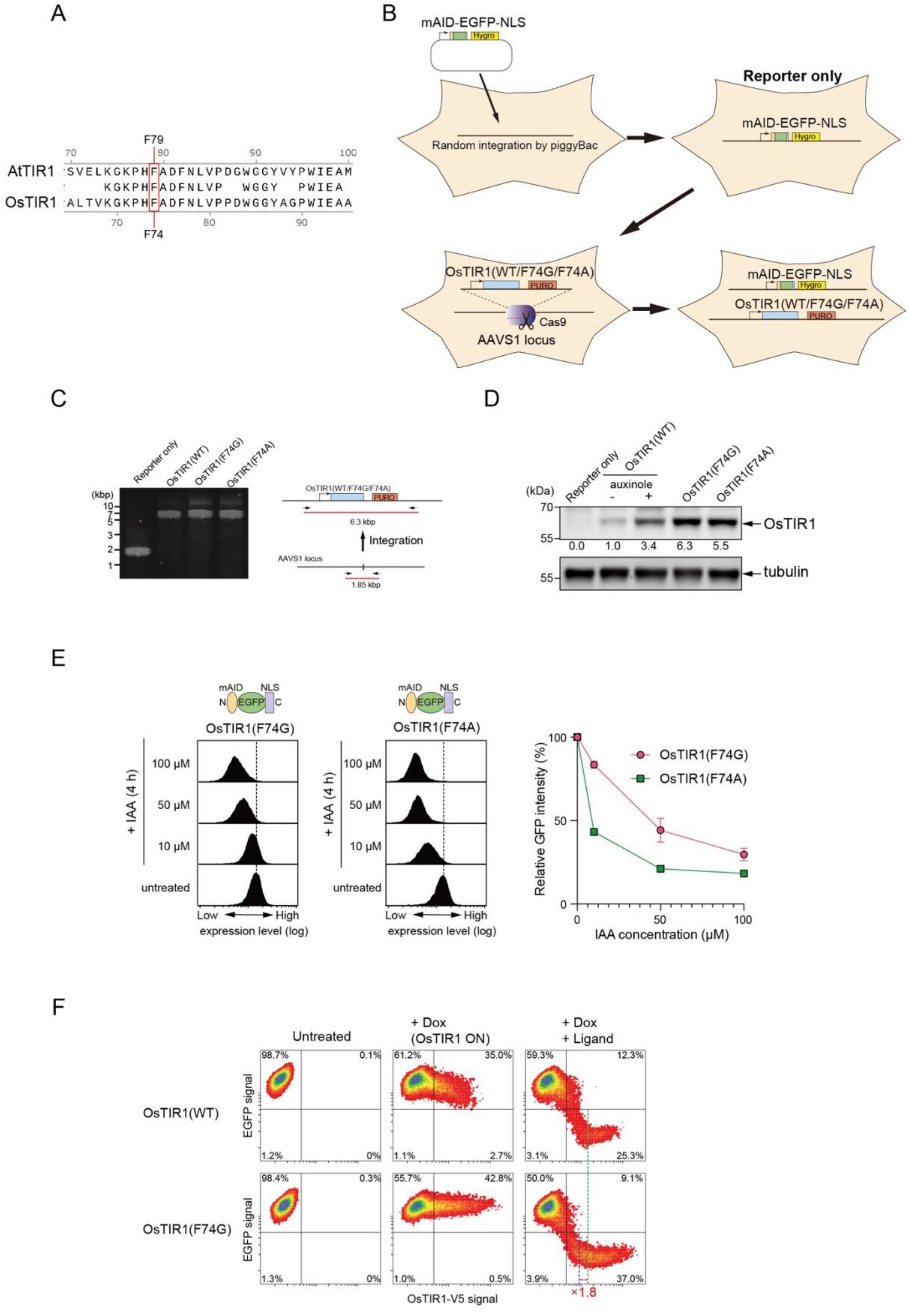

Supplementary Figure 1

Related to Figure 1. (**A**) Comparison of the site for introducing a mutation in AtTIR1 and OsTIR1. (**B**) Schematic illustration showing the strategy for generating HCT116 cell lines expressing a mAID-EGFP-NLS reporter and OsTIR1(WT, F74G, or F74A). (**C**) Confirmation of bi-allelic insertion of OsTIR1(WT, F74G, or F74A) at the AAVS1 locus. The indicated primers shown as arrows (5'-CACTTTGAGCTCTACTGGCTTCTGC-3' and 5'-CCACCCAAAAGGCAGCCTGGTAGAC-3') were used for genomic PCR. We repeated this experiments twice and obtained consistent results. (**D**) Western blotting showing the expression level of OsTIR1(WT), OsTIR1(F74G), and OsTIR1(F74A). To suppress the activity of OsTIR1(WT), 200 μM auxinole was added to the culture for 4 h. The quantified signals relative to OsTIR1(WT) were indicated. We repeated this experiment three times and obtained similar results. (**E**) Reactivity of OsTIR1(F74G) and OsTIR1(F74A) to IAA. The cells were treated with the indicated concentrations of IAA for 4 h. The reporter expression was detected by flow cytometry (left) and quantified data are shown on the right. Data are presented as mean values ± SD (n = 3 independent experiments). (**F**) Two reporter HCT116 lines similar to those shown in panel A were generated. In these cells, the expression of OsTIR1(WT or F74G)-V5 was driven by a conditional tetracycline-inducible promoter. To induce reporter degradation, 0.5 μg/mL of doxycycline was added for 24 h. Subsequently, 100 μM IAA or 1 μM 5-Ph-IAA was added to OsTIR1(WT) or OsTIR1(F74G) expressing cells, respectively. The cells were stained with anti-V5 antibody before flow cytometric analysis.

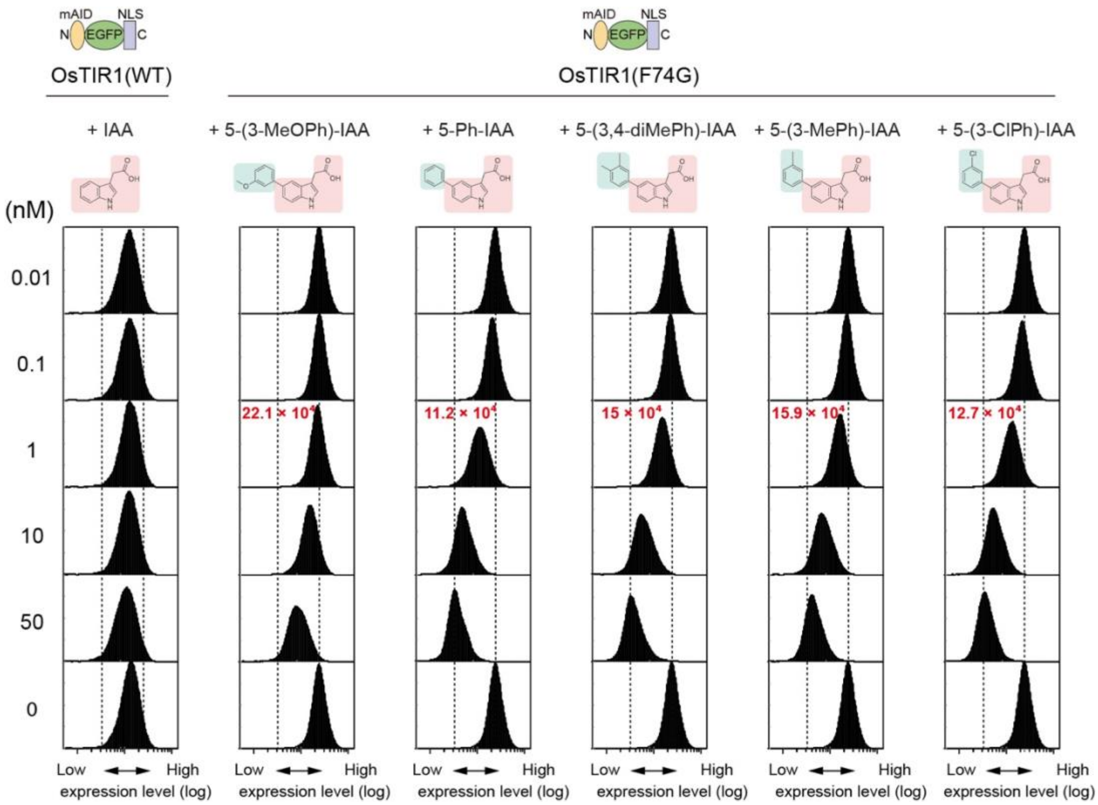

Supplementary Figure 2

Related to Figure 1. Screening to identify an effective bumped-IAA analogue. Indicated ligands were added to cells expressing a mAID-EGFP-NLS reporter with OsTIR1(WT or F74G) for 4 h. The median values at 1 nM are also indicated (arbitrary units).

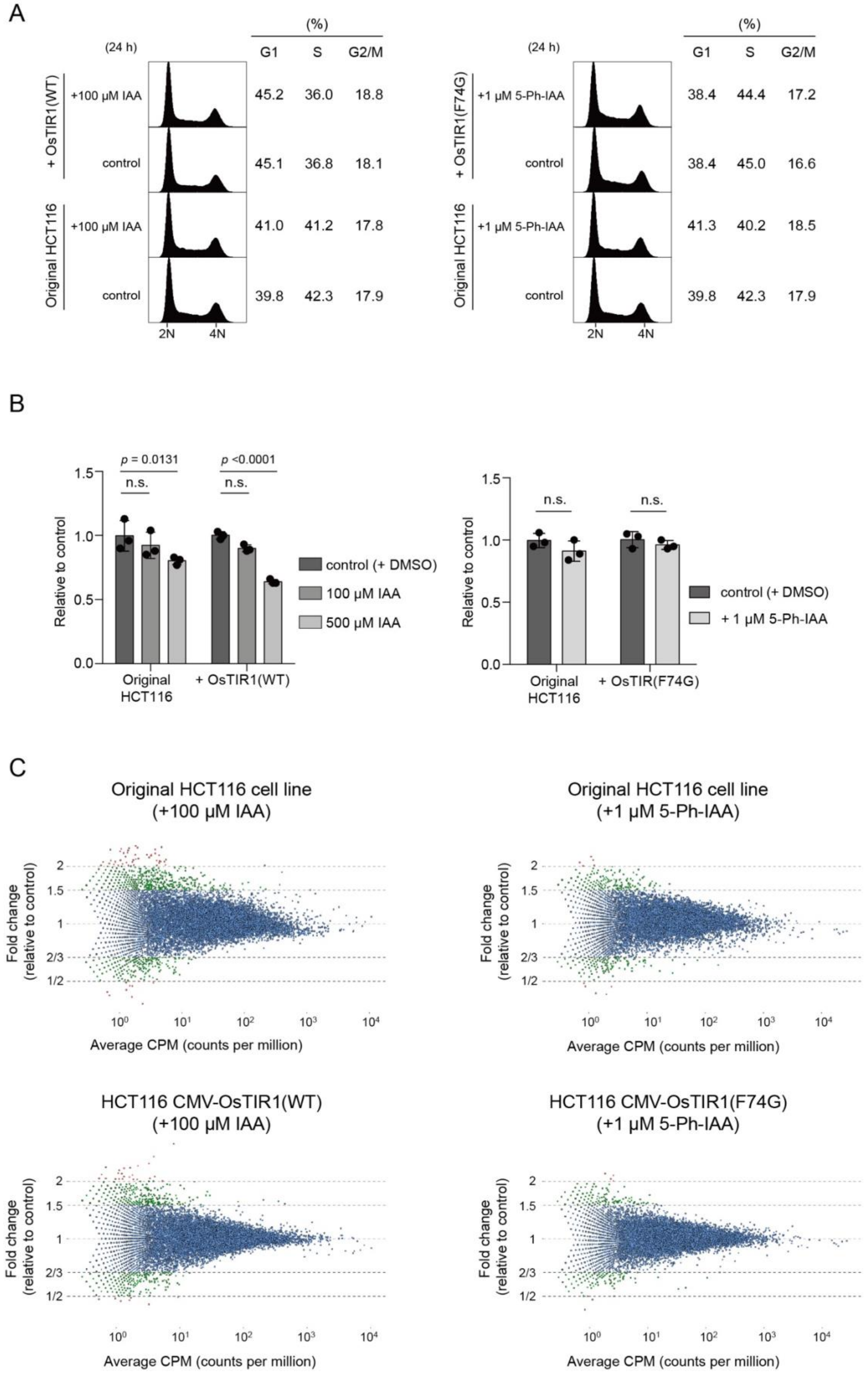

Supplementary Figure 3

Related to Figure 1. Treatment with 1 μM 5-Ph-IAA shows less side effect than that with 100 μM IAA. (**A**) Cell cycle distribution after treatment with DMSO (control), 100 μM IAA, or 1 μM 5-Ph-IAA for 24 h. The original HCT116 cells and those expressing OsTIR1(WT or F74G) were subjected. (**B**) Colony formation efficiency of the original HCT116 cells and those expressing OsTIR1(WT or F74G) in the presence of DMSO (control), IAA, or 5-Ph-IAA. Data are presented as mean values ± SD (*n* = 3 independent experiments, two-way ANOVA). (**C**) Plot depicting the $\log_2$-transformed fold change in gene expression in relation to the average of the normalized counts for each gene in the original HCT116 cells and those expressing OsTIR1(WT or F74G) after treatment with DMSO (control), 100 μM IAA, or 1 μM 5-Ph-IAA for 24 h.

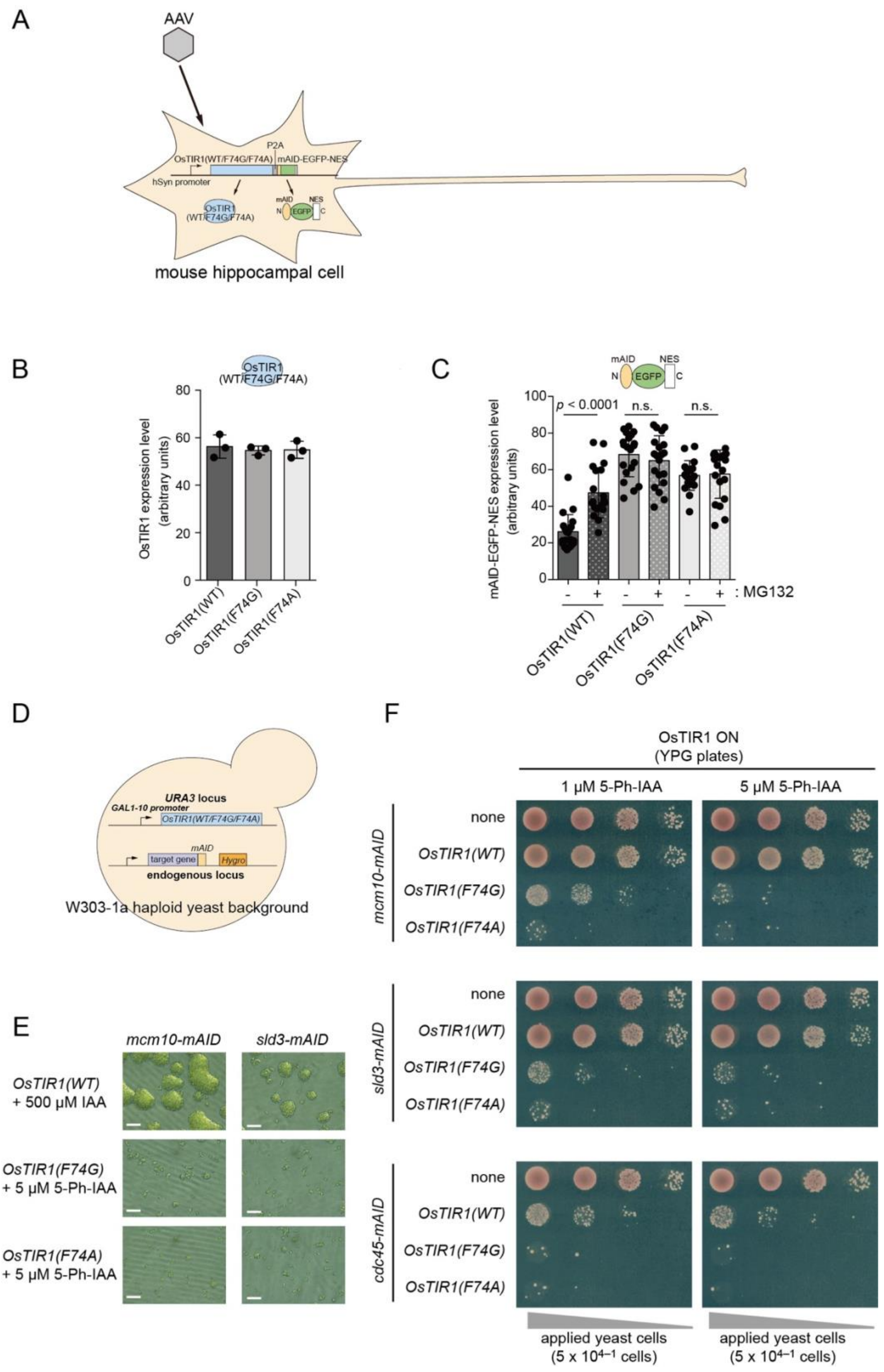

Supplementary Figure 4

Related to Figure 2. (**A**) Schematic illustration showing the strategy for introducing a mAID-EGFP-NES reporter and OsTIR1(WT, F74G, or F74A) using AAV. (**B**) The

expression level of OsTIR1 in neurons after AAV infection. The cells were immuno-stained using anti-OsTIR1 antibody and stained cells were quantified. Data are presented as mean values ± SD (*n* = 3 independent experiments). (**C**) The initial expression level of the reporter in the transfected neurons. A proteasome inhibitor, MG132, was added at 1 μM for 2 h before image acquisition. Data are presented as mean values ± SD (*n* = 20 cells examined, two-tailed t-test). (**D**) Schematic illustration showing the strategy to generate yeast mutants. (**E**) Microscopic pictures showing *mcm10-mAID* and *sld3-mAID* mutants shown in Figure 2C. Scale bars show 100 μm. We repeated this experiment twice and obtained similar results. (**F**) Indicated yeast mutants were spotted on a YPG plate containing 1 or 5 μM 5-Ph-IAA. Plates were incubated at 25°C for three days.

A

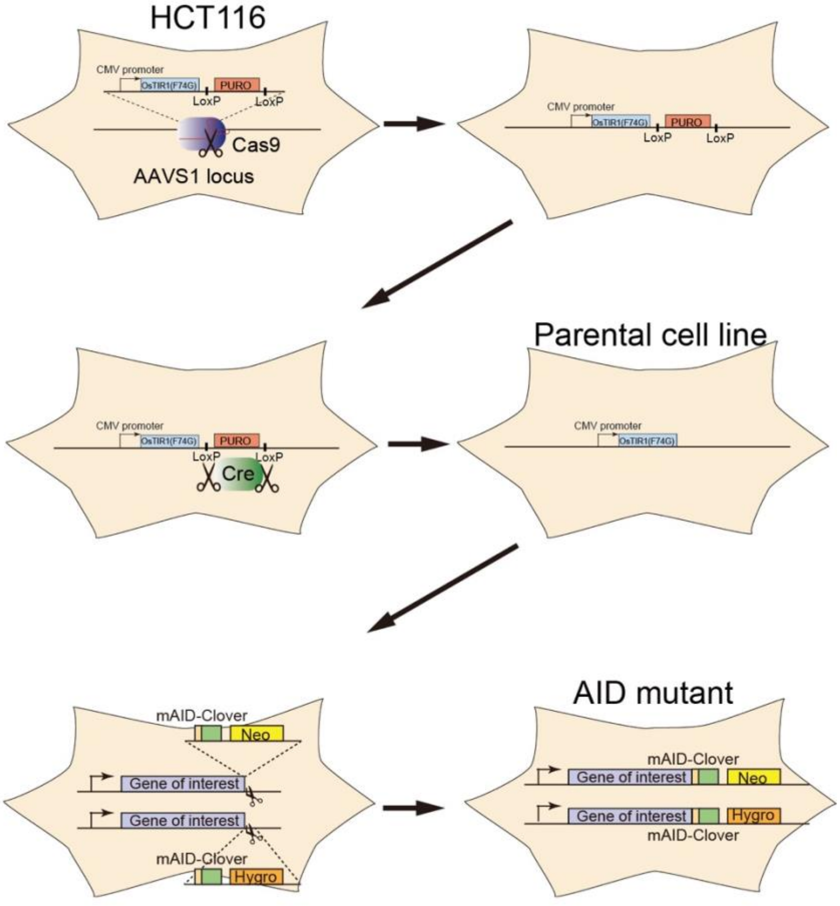

B

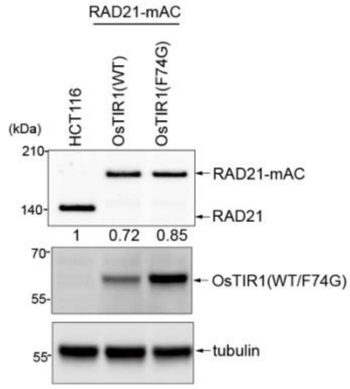

Supplementary Figure 5

Related to Figures 3 and 4. (**A**) Schematic illustration showing a strategy for generating conditional cell lines using the AID2 system. (**B**) The initial expression level of RAD21-mAC and OsTIR1. RAD21 and OsTIR1 were detected by using anti-RAD21 and -OsTIR1 antibodies, respectively. Tubulin is a loading control. RAD21

bands were quantified and these values are shown below the RAD21 blot. We repeated this experiment three times and obtained similar results.

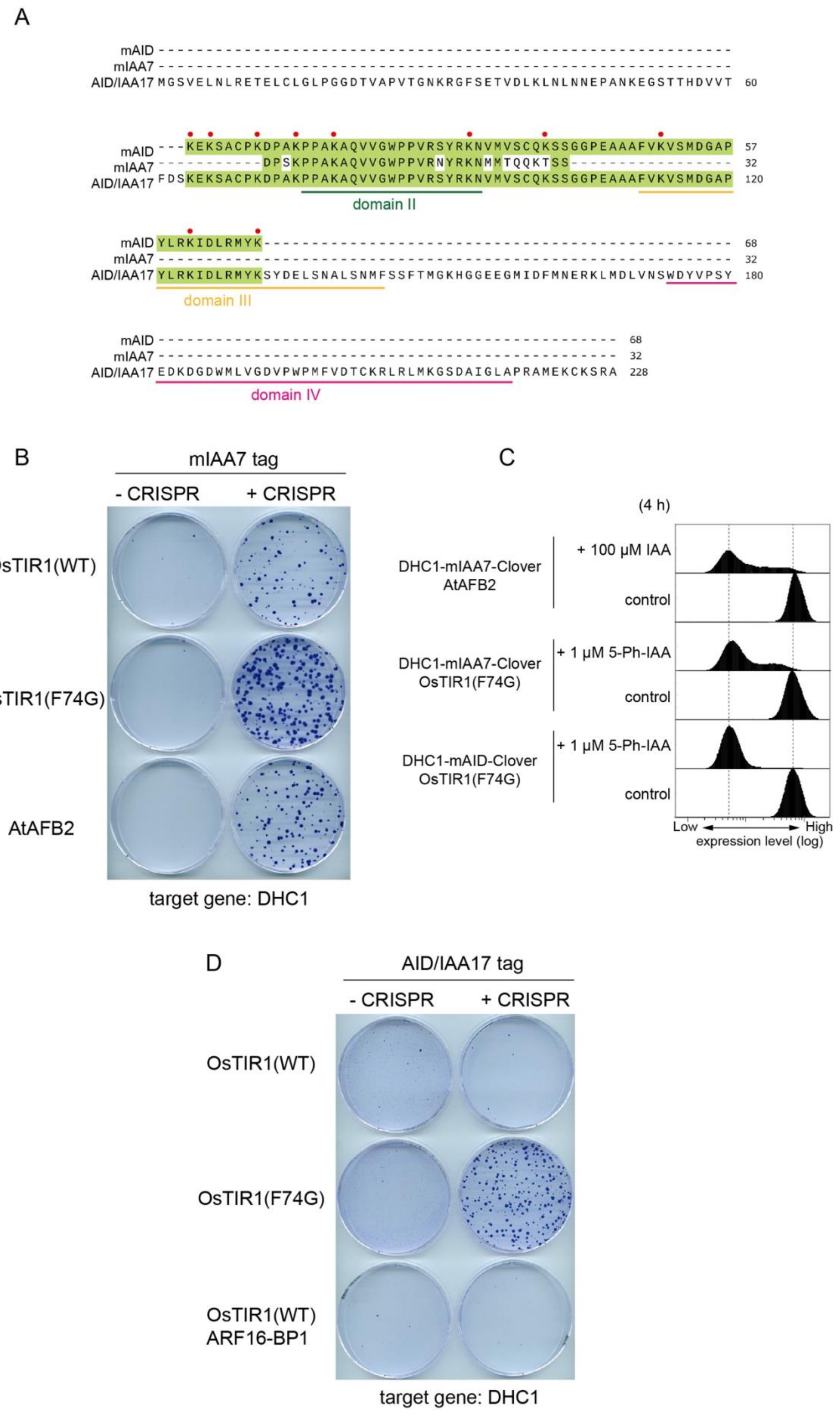
A
mAID
mIAA7
AID/IAA17 MGSVELNLRETELCLGLPGGDTVAPVTGNKRGFSETVDLKLNLNNEPANKEGSTTHDVVT 60
mAID KEKSACPKDPAKPPAKAQVVGWPPVRSYRKNVMVSCQKSSGGPEAAAFVKVSMDGAP 57
mIAA7 DPSKPPAKAQVVGWPPVRNYRKNMMTQQKTSS 32
AID/IAA17 FDSKEKSACPKDPAKPPAKAQVVGWPPVRSYRKNVMVSCQKSSGGPEAAAFVKVSMDGAP 120
domain II
mAID YLRKIDLRMYK 68
mIAA7 32
AID/IAA17 YLRKIDLRMYKSYDELSNALSNMFSSFTMGKHGGEEGMIDFMNERKLMDLVNSWDYVPSY 180
domain III
mAID 68
mIAA7 32
AID/IAA17 EDKDGDWMLVGDVPWPMFVDTCKRLRLMKGSDAIGLAPRAMEKCKSRA 228
domain IV
B
mIAA7 tag
- CRISPR
+ CRISPR
OsTIR1(WT)
OsTIR1(F74G)
AtAFB2
target gene: DHC1
C
(4 h)
DHC1-mIAA7-Clover
AtAFB2
+ 100 μM IAA
control
DHC1-mIAA7-Clover
OsTIR1(F74G)
+ 1 μM 5-Ph-IAA
control
DHC1-mAID-Clover
OsTIR1(F74G)
+ 1 μM 5-Ph-IAA
control
Low
High
expression level (log)
D
AID/IAA17 tag
- CRISPR
+ CRISPR
OsTIR1(WT)
OsTIR1(F74G)
OsTIR1(WT)
ARF16-BP1
target gene: DHC1

Supplementary Figure 6

Related to Figure 4. A comparison of the AID2 system to the AtAFB2-mIAA7 and ARF-AID systems. (**A**) Amino-acid sequence alignment of mAID, mIAA7, and AID/IAA17. Red dots show lysine residues within mAID. The domain II is essential for OsTIR1 binding. The domains III and IV are required for complex formation with ARF16-PB1. (**B**) Colony formation after transfection to fuse the mIAA7 tag to DHC1 in the indicated backgrounds. The DHC1 tagging donors containing the mIAA7 tag were transfected with or without a CRISPR–Cas9 plasmid. Colonies were formed in the presence of 700 μg/ml of Neomycin and 100 μg/ml of Hygromycin for 11 days. After double selection, colonies were stained with crystal violet. (**C**) Optimal degradation is achieved with the OsTIR1(F74G)–mAID pair. FACS profiles showing DHC1-mAC signal histogram before and after 4 h treatment with 100 μM IAA or 1 μM 5-Ph-IAA. (**D**) Colony formation after transfecting to fuse the AID/IAA17 tag to DHCT1 in the indicated backgrounds. The DHC1 tagging donors containing the AID/IAA17 tag were transfected with or without a CRISPR–Cas9 plasmid. Colonies were formed in the presence of 700 μg/ml of Neomycin and 100 μg/ml of Hygromycin for 10 days. After double selection, colonies were stained with crystal violet.

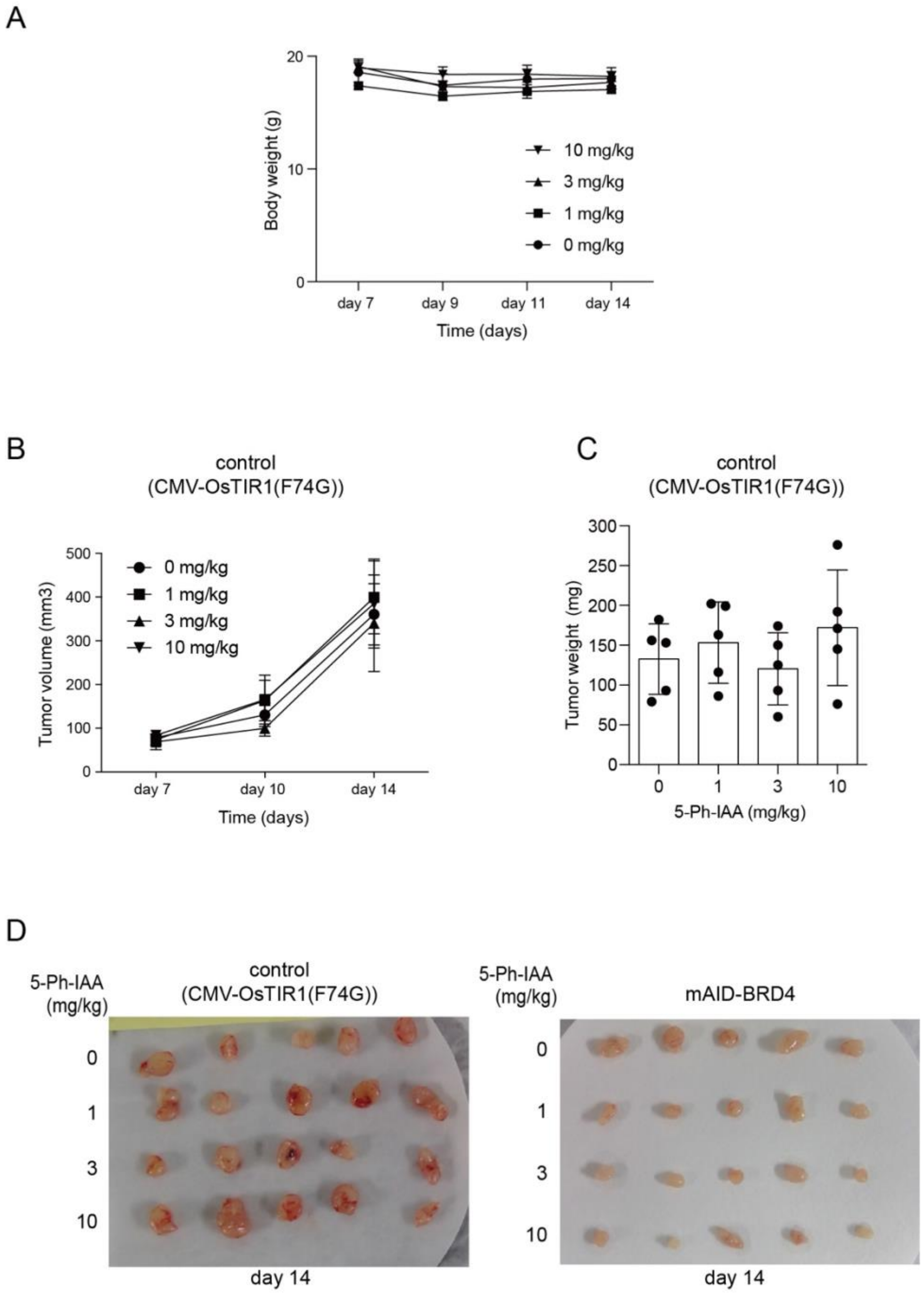

Supplementary Figure 7

Related to Figure 5. (**A**) Graph showing the body weight of control mice. Mice without xenograft transplantation were treated with the indicated doses of 5-Ph-IAA. Data are presented as mean values ± SD (*n* = 4 animals). (**B**) Tumour volume of control xenograft. HCT116 cells expressing OsTIR1(F74G) were transplanted and the mice were treated with the indicated dose of 5-Ph-IAA as in Figure 5B. Data are presented as mean values ± SD (*n* = 5 animals). (**C**) Tumour weight of control xenograft on day 14. HCT116 cells expressing OsTIR1(F74G) were transplanted and

the mice were treated with the indicated dose of 5-Ph-IAA as in Figure 5B. Data are presented as mean values ± SD (*n* = 5 animals). (**D**) Control xenograft tumours (left) and mAC-BRD4 xenograft tumours (right) on day 14.

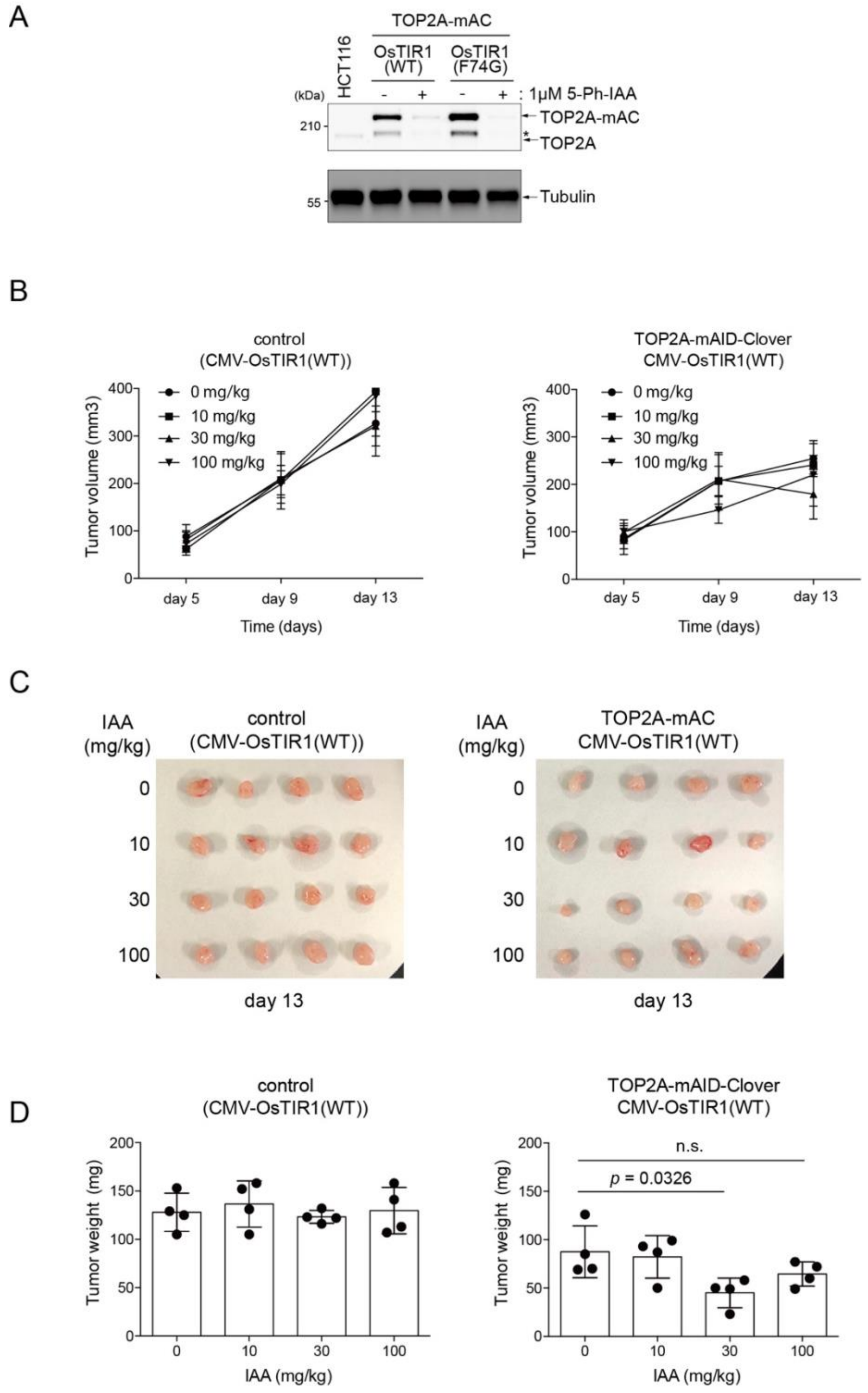

Supplementary Figure 8

Related to Figure 5. (**A**) TOP2-mAID-Clover (TOP2-mAC) cells expressing OsTIR1(WT or F74G) were mock-treated with DMSO or treated with the indicated ligand for 4 h. Proteins were separated and blotted using anti-TOP2 antibody. Tubulin is a loading control. The asterisk shows a partial degradation product of TOP2A-mAC. We repeated this experiment twice and obtained similar results. (**B**)

Tumour volume of control and TOP2-mAC xenografts using the original AID system. Mice were treated with the indicated dose of IAA as in Figure 5B. Data are presented as mean values ± SD (*n* = 4 animals). (**C**) Control xenograft tumours on day 13. (**D**) Tumour weight of control xenograft on day 13. Mice were treated with the indicated dose of IAA as in Figure 5B. Data are presented as mean values ± SD (*n* = 4 animals, two-tailed t-test).

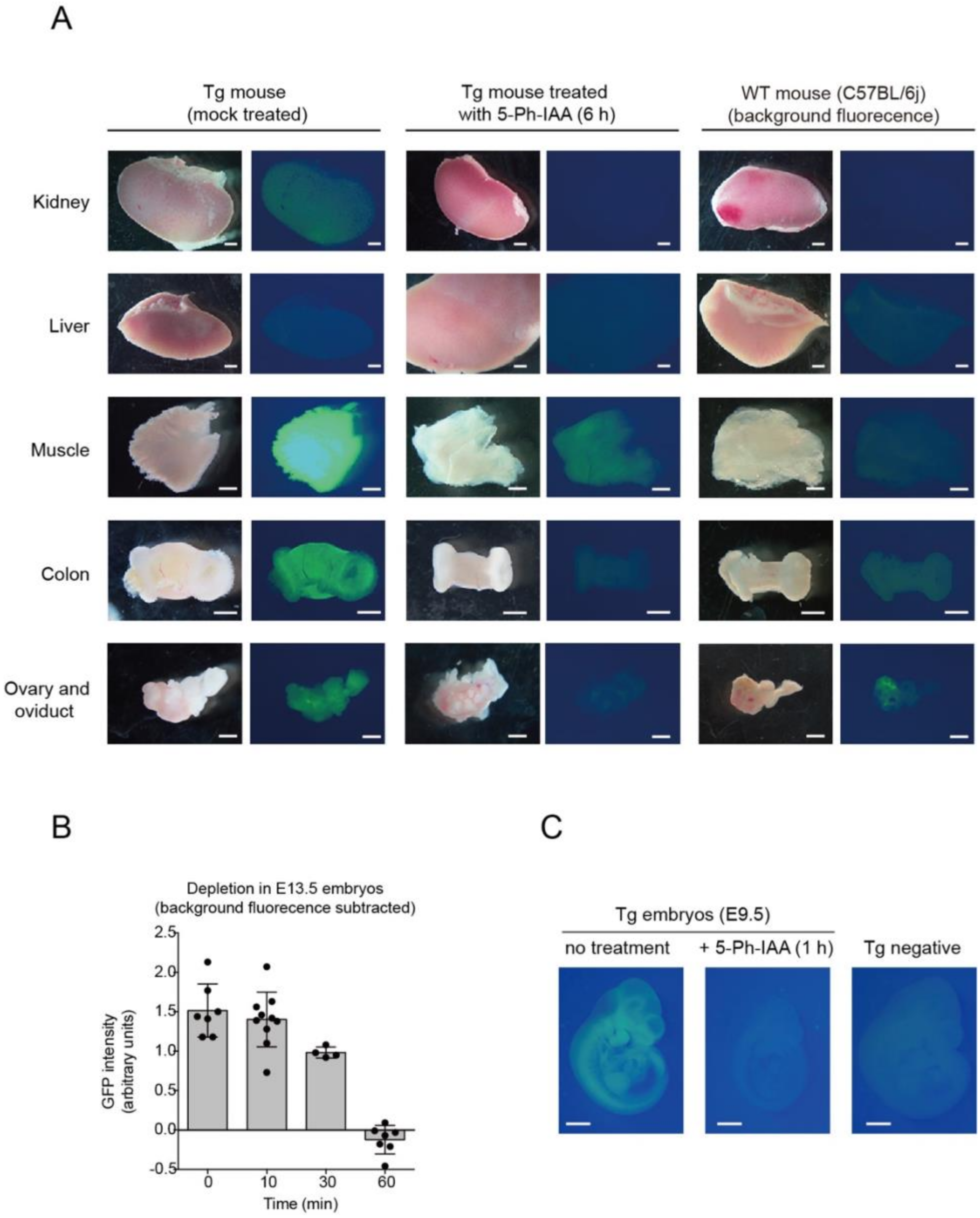

Supplementary Figure 9

Related to Figure 6. (**A**) Comparison of the indicated organs removed from 5-Ph-IAA-treated Tg #1 mice. The organs derived from the wild-type mouse show background fluorescence. Scale bars show 1 mm. (**B**) GFP intensity of the data shown in Figure 6E was quantified. The average GFP intensity of Tg-negative embryos was subtracted from that of Tg-positive littermates. Data are presented as mean values ± SD and the sample number is shown as dots (*n* = 4 to 10 embryos). (**C**) Representative images of E9.5 embryos used for western blot shown in Figure 6F. Scale bar shows 500 μm. We repeated this experiment twice and obtained similar results.

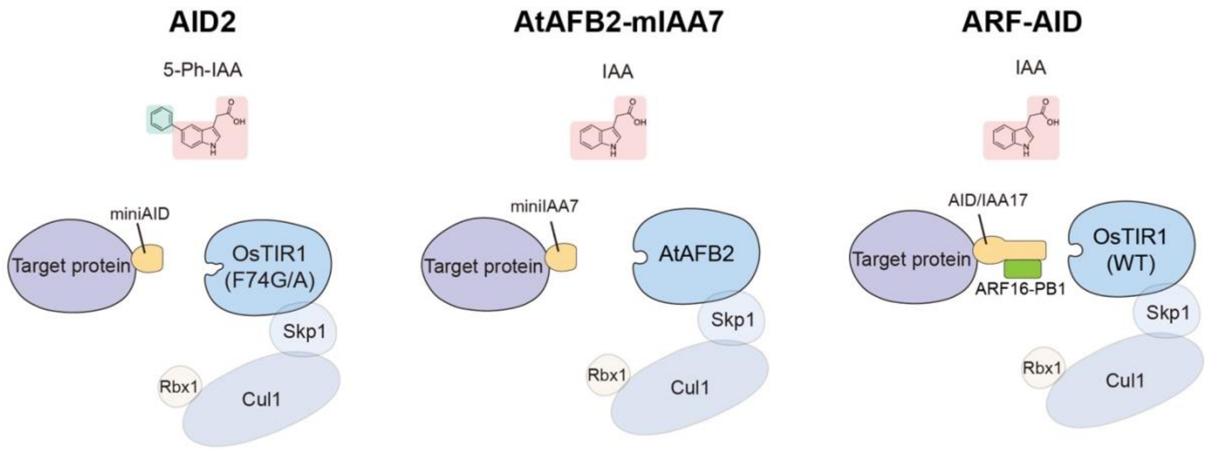

	AID2	AtAFB2-mIAA7	ARF-AID
Components	OsTIR1(F74G/A)<br>miniAID	AtAFB2<br>miniIAA7	OsTIR1(WT)<br>AID/IAA17<br>ARF16-PB1
Size of degron	7.4 kDa	3.6 kDa	26.4 kDa
Ligand	< 1 μM 5-Ph-IAA	100–500 μM IAA	100–500 μM IAA
Publication	This study	Li et al. Nat Meth 2019	Sathyan et al. G&D 2019
Note	Two-component system	Two-component system	Three-component system, which works only with AID. Not compatible with miniAID and miniIAA7.

Supplementary Figure 10

Comparison of AID2, AtAFB2-mIAA7, and ARF-AID systems. The schematic illustrations show required components and their activating ligand. The table shows their features and other information.

Supplementary Table 1

A) Number of genes affected by 100 μM IAA or 1 μM 5-Ph-IAA treatment.

Ligand	Cell line	> 2	> 1.5	< 2/3	< 1/2
+ 100 μM IAA	HCT116	55	791	316	11
	HCT116 OsTIR1(WT)	29	488	381	7
+ 1 μM 5-Ph-IAA	HCT116	9	265	200	5
	HCT116 OsTIR1(F74G)	4	195	162	5

B) Comparison of affected genes in HCT116

Cell line	Ligand	Number of genes		OR	95% CI	*P*-value
		Affected	Unaffected			
HCT116	+ 100 μM IAA	1173	17284	2.29	2.05-2.56	3.6×10^{-55}
HCT116	+ 1μM 5-Ph-IAA	479	16179	Reference	NA	NA

* OR, odds ratio; CI, confidence interval; and NA, not available.
† The proportion of genes affected by 100 μM IAA treatment was significantly larger than that by 1 μM 5-Ph-IAA treatment.

C) Comparison of affected genes in HCT116 OsTIR1 (WT) and OsTIR1(F74G)

Cell line	Ligand	Number of genes		OR	95% CI	*P*-value
		Affected	Unaffected			
HCT116 OsTIR1(WT)	+ 100 μM IAA	905	17267	2.35	2.07-2.67	7.1×10^{-46}
HCT116 OsTIR1(F74G)	+ 1μM 5-Ph-IAA	366	16442	Reference	NA	NA

* OR, odds ratio; CI, confidence interval; and NA, not available.
† The proportion of genes affected by the AID system was significantly larger than that by the AID2 system.

Supplementary Table 2
List of plasmids used in this study.

Figures	Plasmid name	Description	Addgene ID
F1B–E, and SF1B	pAY5	mAID-EGFP-NLS piggyBac	140532
F1B–E, SF1B	pMK364	AAVS1 CMV-OsTIR1(WT)	121184
F1B–E, SF1B and SF5	pMK381	AAVS1 CMV-OsTIR1(F74G)	140536
F1B–E, SF1B	pMK395	AAVS1 CMV-OsTIR1(F74A)	159992
F1B–E, SF1B and SF5	AAVS1 T2 CRISPR	AAVS1 CRISPR	72833
SF1F	AAVS1-Tet-OsTIR1(WT)-V5	AAVS1-Tet-OsTIR1(WT)-V5	158663
SF1F	AAVS1-Tet-OsTIR1(F74G)-V5	AAVS1-Tet-OsTIR1(F74G)-V5	158664
F2A, B and SF4A	pAAV-hSyn-OsTIR1(WT)	OsTIR1(WT)-P2A-mAID-EGFP-NES	140729
F2A, B and SF4A	pAAV-hSyn-OsTIR1(F74G)	OsTIR1(F74G)-P2A-mAID-EGFP-NES	140730
F2A, B and SF4A	pAAV-hSyn-OsTIR1(F74A)	OsTIR1(F74A)-P2A-mAID-EGFP-NES	140731
F2C and SF4D	pMK198	GAL-OsTIR1(WT)	140655
F2C and SF4D	pMK419	GAL-OsTIR1(F74G)	140656
F2C and SF4D	pMK425	GAL-OsTIR1(F74A)	140657
F3	pMK262	RAD21-mAC Neo donor	140538
F3	pMK265	RAD21-mAC Hygro donor	140539
F3	RAD21 C-tag CRISPR	RAD21 tagging CRISPR	140540
F4A and SF6	pMK233	AAVS1 CMV-AtAFB2	140537
F4A–D and SF6	pMK296	DHC1-mAC Neo donor	140541
F4A–D and SF6	pMK297	DHC1-mAC Hygro donor	140542
SF6B and C	DHC1-mIAA7 Neo	DCH1-mIAA7 Neo donor	140543
SF6B and C	DHC1-mIAA7 Hygro	DCH1-mIAA7 Hygro donor	140544
F4A–D and SF6	DHC1 CRISPR	DHC1 tagging CRISPR	140545
SF6D	pMGS46 (Sathyan et al.)	AAVS1 CMV-ARF16-Bp1-P2A-OsTIR1(WT)	126580
SF6D	DHC1-AID-Clover donor (Neo)	DCH1-AID Neo donor	158621
SF6D	DHC1-AID-Clover donor (Hygro)	DCH1-AID Hygro donor	158622
F4E	CTCF-mAC donor (Neo)	CTCF-mAC Neo donor	140645
F4E	CTCF-mAC donor (Hygro)	CTCF-mAC Hygro donor	140646
F4E	CTCF-C CRISPR	CTCF tagging CRISPR	140647
F4E	SMC2-mAC donor (Hygro)	SMC2-mAC Hygro donor	140648
F4E	SMC2 CRISPR	CTCF tagging CRISPR	140649
F4E	mAC-POLR2A donor (Hygro)	Hygro mAC-POLR2A donor	124496
F4E	POLR2A-N CRISPR pX330	POLR2A tagging CRISPR	124495
F5	mAID-BRD4 donor	Hygro mAID-BRD4 tagging donor	140650
F5	BRD4-N CRISPR	BRD4-N CRISPR	140651
F5 and SF8	pMK321	TOP2A-mAC Neo donor	140652
F5 and SF8	pMK322	TOP2A-mAC Hygro donor	140653
F5 and SF8	pMK312	TOP2A tagging CRISPR	140654
F6	pMK427	OsTIR1(WT)-P2A-mAID-EGFP-Nluc TOL2	140658
F6	pMK411	OsTIR1(F74G)-P2A-mAID-EGFP-Nluc TOL2	140659

Supplementary Table 3
List of HCT116 cell lines used in this study.

Cell line	Genotype	Publication
Reporter cell line	mAID-EGFP-NLS (reporter only)	This study (F1, SF1)
Reporter line with OsTIR1(WT)	mAID-EGFP-NLS, CMV-OsTIR1(WT)	This study (F1, SF1,2)
Reporter line with OsTIR1(F74G)	mAID-EGFP-NLS, CMV-OsTIR1(F74G)	This study (F1, SF1, 2, 3)
Reporter line with OsTIR1(F74A)	mAID-EGFP-NLS, CMV-OsTIR1(F74A)	This study (F1, SF1, 2)
Reporter line with Tet-OsTIR1(WT)	mAID-EGFP-NLS, Tet-OsTIR1(WT)	This study (SF1)
Reporter line with Tet-OsTIR1(F74G)	mAID-EGFP-NLS, Tet-OsTIR1(F74G)	This study (SF1)
OsTIR1(WT)	CMV-OsTIR1(WT)	This study (SF 3, 8)
OsTIR1(F74G)	CMV-OsTIR1(F74G)	This study (SF 3, 7)
RAD21-mAC OsTIR1(WT)	RAD21-mAID-Clover, CMV-OsTIR1(WT)	Natsume et al. Cell Reports, 2016 (F3, SF 5)
RAD21-mAC OsTIR1(F74G)	RAD21-mAID-Clover, CMV-OsTIR1(F74G)	This study (F3, SF 5)
DHC1-mAC OsTIR(F74G)	DHC1-mAID-Clover, CMV-OsTIR1(F74G)	This study (F3, SF 5)
AtAFB2	CMV-AtAFB2	Natsume et al. Genes & Development, 2017 (F4, SF6)
DHC1-mAC AtAFB2	DHC1-mAID-Clover, CMV-AtAFB2	This study (F4, SF 6)
DHC1-mIAA7 AtAFB2	DHC1-mIAA7-Clover, CMV-AtAFB2	This study (SF 6)
OsTIR1(WT) ARF16-BP1	OsTIR1(WT)-P2A-ARF16-BP1	This study (SF6)
SMC2-mAC	SMC2-mAID-Clover, CMV-OsTIR1(F74G)	This study (F4)
mAC-POLR2A	mAID-Clover-POLR2A, CMV-OsTIR1(F74G)	This study (F4)
CTCF-mAC	CTCF-mAID-Clover, CMV-OsTIR1(F74G)	This study (F4)
mAID-BRD2	mAID-BRD4, CMV-OsTIR1(F74G)	This study (F5)
TOP2A-mAC OsTIR1(F74G)	TOP2A-mAID-Clover, CMV-OsTIR1(F74G)	This study (F4, SF8)
TOP2A-mAC OsTIR1(WT)	TOP2A-mAID-Clover, CMV-OsTIR1(WT)	This study (SF8)

Supplementary Methods

Chemical synthesis of ligands

1. General experimental condition.

^{1}H- and ^{13}C-NMR spectra were recorded on a JEOL ECS400 NMR spectrometer (JEOL, Japan). Peak multiplicities are quoted in Hz. Mass spectra were measured on a JMS-700 spectrometer (JEOL, Japan). Column chromatography was performed with Merck silica gel 60 (230–400 mesh, Merck, Japan). All chemicals were purchased from Tokyo Chemical Industry Japan (Tokyo, Japan) and Sigma-Aldrich Japan (Tokyo, Japan) unless otherwise stated. 5-Ph-IAA is commercially available as a reagent (BioAcademia #30-003).

2. Synthesis of 5-Ph-IAA (5-phenyl-indole-3-acetic acid)

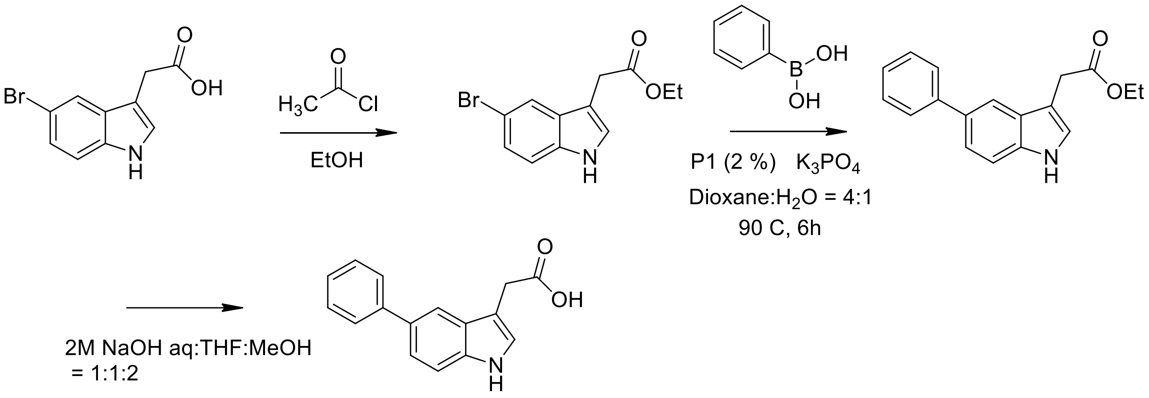

To the solution of 5-bromoindole-3-acetic acid (508 mg, 2.0 mmol) in ethanol (25 mL) was added dropwise acetyl chloride (1 mL), and then stirred for 3h at room temperature. The reaction mixture was added to water (100 mL), and extracted with EtOAc (50 mL × 3). The organic layer was washed with brine, and then dried over Na_2SO_4. 5-Bromoindole-3-acetic acid ethyl ester was obtained as a brown solid (556 mg, 99% yield). ^{1}H-NMR (400 MHz, $CDCl_3$) δ 8.23 (s, 1H), 7.73 (s, 1H), 7.24 (dd, J = 8.5, 2.1 Hz, 1H), 7.15 (d, J = 8.7 Hz, 1H), 7.07 (s, 1H), 4.18 (q, J = 7.2 Hz, 2H), 3.71 (s, 2H), 1.28 (t, J = 7.3 Hz, 3H), ^{13}C-NMR (100 MHz, $CDCl_3$) δ 172.07, 134.82, 129.05, 125.08, 124.49, 121.64, 112.99, 112.77, 108.21, 61.11, 31.31, 14.32; FAB-MS *m/z* 282 $[M+H]^+$. This ethyl ester was used for the reaction without further purification.

5-Bromoindole-3-acetic acid ethyl ester (500 mg, 1.8 mmol), tripotassium phosphate (752 mg, 3.6 mmol) and phenylboronic acid (325 mg, 2.7 mmol) were dissolved in dioxiane-H_2O (4:1, 20 mL) and then the catalyst P1, 14 mg of XPhos-Pd-G2 (Sigma-Aldrich, #741825), was added. The mixture was stirred for 6 h at 90°C[10]. The reaction mixture was poured into water (50 mL) and extracted with EtOAc (60 mL × 3). The organic layer was washed with brine. After dried over Na_2SO_4, the solvent was removed *in vacuo*. The residue was purified by a silica gel column chromatography (hexane : EtOAc = 3:1) to give a 5-phenyl-indole-3-acetic

acid ethyl ester as pale yellow oil (454 mg, 92% yield). ^{1}H-NMR (400 MHz, $CDCl_3$) δ 8.17 (s, 1H), 7.84 (s, 1H), 7.68-7.66 (m, 2H), 7.47-7.43 (m, 3H), 7.37 (d, J = 8.2 Hz, 1H), 7.32 (t, J = 7.3 Hz, 1H), 7.14 (s, 1H), 4.19 (q, J = 7.0 Hz, 2H), 3.82 (s, 2H), 1.28 (t, J = 7.1 Hz, 3H), ^{13}C-NMR (100 MHz, $CDCl_3$) δ 172.12, 142.49, 135.58, 133.18, 128.59, 127.68, 127.37, 126.29, 123.78, 121.99, 117.34, 111.40, 108.80, 60.82, 31.34, 14.21); FABMS *m/z* 280 $[M+H]^+$.

The ethyl ester (322 mg, 1.15 mmol) was hydrolyzed in 2 M NaOH aq-THF-MeOH (1:1:2, 12 mL) for 4 h at room temperature. The reaction mixture was acidified by 6 M HCl and then extracted with EtOAc (50 mL × 3). The organic layer was washed with brine, and then dried over Na_2SO_4. The residue was purified by a silica gel column chromatography ($CHCl_3$: acetone = 5:1) to yield a 5-phenyl-indole-3-acetic acid as crystal (208 mg, yield 72%). ^{1}H-NMR (400 MHz, acetone-d_6) δ10.61 (s, 1H), 10.17 (s, 1H), 7.89 (s, 1H), 7.68-7.65 (m, 2H), 7.48-7.46 (m, 1H), 7.44-7.40 (m, 3H), 7.34 (d, J = 2.3 Hz, 1H), 7.26 (tt, J = 7.3, 1.4 Hz, 1H), 3.82 (s, 2H), ^{13}C-NMR (100 MHz, acetone-d_6) δ 173.20, 143.65, 137.15, 133.06, 129.51, 129.15, 127.86, 126.96, 125.39, 121.85, 118.07, 112.54, 109.65, 31.39; FABMS *m/z* 252 $[M+H]^+$.

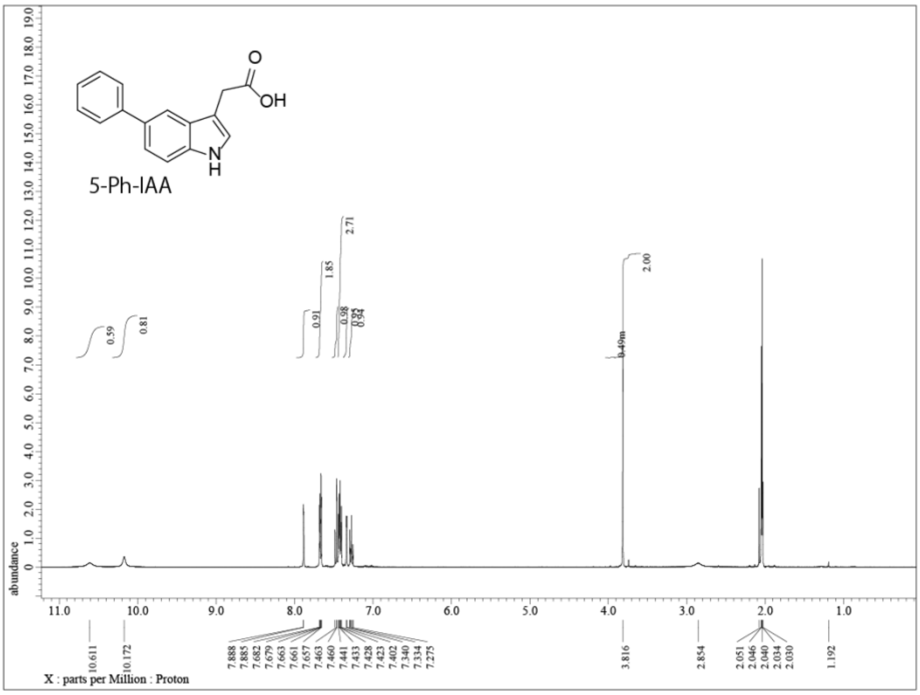

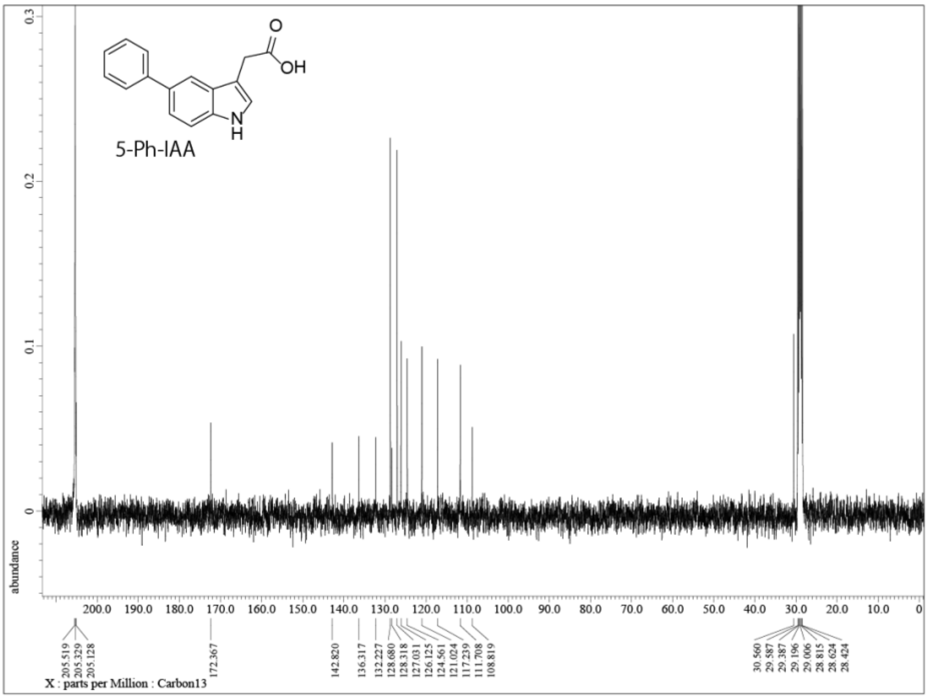

3. **Synthesis of 5-(3-methylphenyl)-indole-3-acetic acid, 5-(3,4-dimethylphenyl)-indole-3-acetic acid and 5-(3-chlorophenyl)-indole-3-acetic acid**

5-Bromoindole-3-acetic acid ethyl ester (0.4 mmol), $PdCl_2(PPh_3)_2$ (15 mg, 0.02 mmol) and the corresponding arylboronic acid (0.8 mmol) were dissolved in DMF-EtOH (1:1, 2 mL) and then 3 M K_2CO_3 aq (0.5 mL) was added. The reaction mixture was refluxed for 5 h at 120°C. The reaction mixture was poured into water (20 mL) and extracted with EtOAc (15 mL × 3). The organic layer was washed with brine. After dried over Na_2SO_4, the solvent was removed *in vacuo*. The residue was purified by a silica gel column chromatography (hexane : EtOAc = 3:1) to give a 5-arylindole-3-acetic acid ethyl ester. 5-Arylindole-3-acetic acid ethyl ester (0.1 - 0.14 mmol), THF (0.25 mL), MeOH (0.25 mL) and 2 M KOH aq (0.25 mL) were added into a screw cap glass vial, and the reaction mixture was then stirred for 2 h at room temperature. The reaction mixture was acidified by 2 M HCl (1 mL), and extracted with EtOAc (2 mL × 3). The organic layer was washed with brine and then dried over Na_2SO_4. The residue was purified by a silica gel column chromatography ($CHCl_3$: acetone = 95:5) to give a 5-arylindole-3-acetic acid.

5-(3-methylphenyl)-indole-3-acetic acid ethyl ester

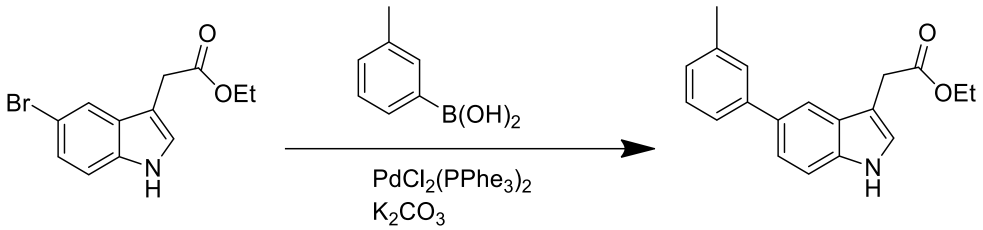

5-(3-methylphenyl)-indole-3-acetic acid ethyl ester was synthesized as a pale brown oil (17 mg, 0.058 mmol, yield 15%) from 5-bromoindole-3-acetic acid (112 mg, 0.40 mmol).^{1}H-NMR (400 MHz, $CDCl_3$) δ 8.10 (s, 1H), 7.81 (s, 1H), 7.49-7.42 (m, 3H), 7.39 (d, J = 8.2 Hz, 1H), 7.32 (t, J=7.8, 1H), 7.19 (d, J = 1.1 Hz, 1H), 7.13 (d, J = 7.3 Hz, 1H), 4.18 (q, J=7.2, 2H), 3.82 (s, 2H), 2.41 (s, 3H), 1.27 (t, J = 7.1 Hz, 3H), ^{13}C-NMR (100MHz, $CDCl_3$) δ 172.12, 142.59, 138.23, 135.68, 133.51, 128.62, 128.32, 127.84, 127.19, 124.61, 123.74, 122.24, 117.51, 111.39, 109.13, 60.91, 31.49, 21.67, 14.34; FABMS *m/z* 294 $[M+H]^+$.

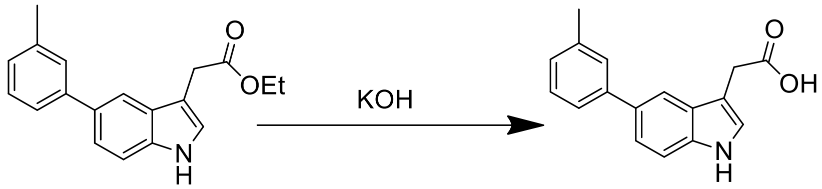

5-(3-methylphenyl)-indole-3-acetic acid was synthesized as a pale-yellow solid (29 mg, 0.11 mmol, yield 80%) from the ester (40 mg, 0.14 mmol). ^{1}H-NMR (400 MHz, $CDCl_3$) δ 8.05 (s, 1H), 7.77 (d, J = 1.4 Hz, 1H), 7.48-7.40 (m, 3H), 7.36 (d, J = 8.7 Hz, 1H), 7.30 (t, J = 7.3 Hz, 1H), 7.16-7.09 (m, 2H), 3.82 (s, 2H), 2.41 (s, 3H), ^{13}C-NMR (100MHz, $CDCl_3$) δ 177.84, 142.51, 138.29, 135.63, 133.70, 128.67, 128.36, 127.69, 127.27, 124.68, 124.04, 122.41, 117.34, 111.53, 108.22, 31.07, 21.68; FABMS *m/z* 266 $[M+H]^+$.

5-(3,4-dimethylphenyl)-indole-3-acetic acid

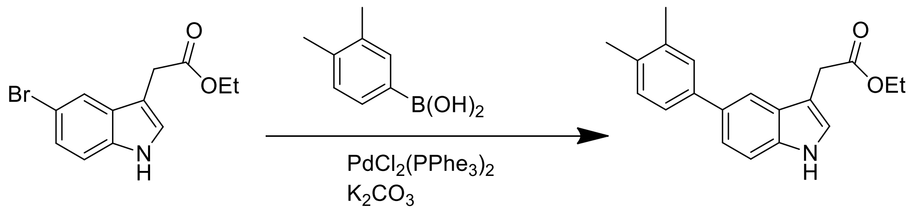

5-(3,4-dimethylphenyl)-indole-3-acetic acid ethyl ester was synthesized as a pale-yellow oil (36 mg, 0.12 mmol, yield 29%) from 5-bromoindole-3-acetic acid (113 mg, 0.40 mmol). ^{1}H-NMR (400 MHz, $CDCl_3$) δ8.10 (s, 1H), 7.79 (d, J = 0.9 Hz, 1H), 7.44 (m, 2H), 7.38 (m, 2H), 7.22-7.14 (m, 2H), 4.17 (q, J = 7.0 Hz, 2H), 3.80 (s, 2H), 2.35 (s, 3H), 2.31 (s,3H), 1.25 (t, J = 7.0 Hz, 3H), ^{13}C-NMR (100MHz, $CDCl_3$) δ 172.17, 140.24, 136.80, 135.55, 134.75, 133.44, 130.30, 128.80, 127.83, 124.85, 123.70, 122.15, 117.23, 111.37, 109.04, 60.89, 31.49, 20.04, 19.46, 14.35; FABMS *m/z* 308 $[M+H]^+$.

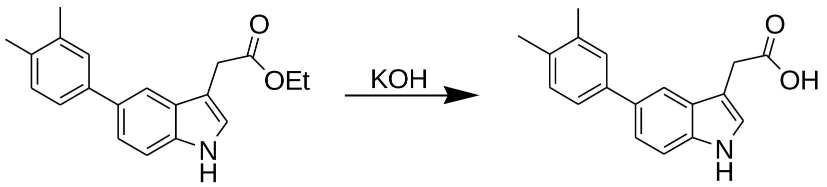

5-(3,4-dimethylphenyl)-indole-3-acetic acid was synthesized as a pale-yellow solid (20 mg, 0.07 mmol, yield 74%) from the ester (30 mg, 0.098 mmol). ^{1}H-NMR (400 MHz, $CDCl_3$) δ 8.06 (s, 1H), 7.76 (s, 1H), 7.46-7.40 (m, 2H), 7.37 (d, J = 8.2 Hz, 2H), 7.22-7.14 (m, 2H), 3.84 (s, 2H), 2.33 (s, 3H), 2.30 (s, 3H),^{13}C-NMR (100MHz, $CDCl_3$) δ 177.32, 140.13, 136.83, 135.50, 134.83, 133.67, 130.05, 128.83, 127.69, 124.89, 123.91, 122.34, 117.10, 111.45, 108.23, 30.99, 20.03, 19.46; FABMS *m/z* 280 $[M+H]^+$.

5-(3-chlorophenyl)-indole-3-acetic acid

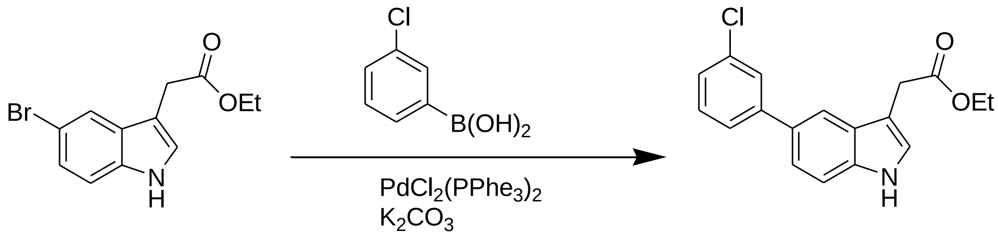

5-(3-chlorophenyl)-indole-3-acetic acid ethyl ester was synthesized as pale-yellow oil (25 mg, 0.80 mmol, yield 20%) from 5-bromoindole-3-acetic acid ethyl ester (113 mg, 0.40 mmol). ^{1}H-NMR (400 MHz, $CDCl_3$) δ 8.14 (s, 1H),7.80 (s, 1H), 7.63 (t, J = 1.8 Hz, 1H), 7.53 (dt, J = 8.0, 1.4 Hz, 1H), 7.42 (d, J = 1.4 Hz, 2H), 7.36 (t, J = 8.0 Hz, 1H), 7.30-7.24 (m, 1H), 7.23 (d, J = 2.3 Hz, 1H), 4.18 (q, J = 7.2 Hz, 2H), 3.81 (s, 2H), 1.27 (t, 7.2 Hz, 3H),^{13}C-NMR (100MHz, $CDCl_3$) δ 171.99, 144.48, 135.96, 134.54, 131.96, 129.90, 127.98, 127.52, 126.38, 125.59, 123.99, 121.98, 117.68, 111.61, 109.29, 60.95, 31.45, 14.34; FABMS *m/z* 314 $[M+H]^+$.

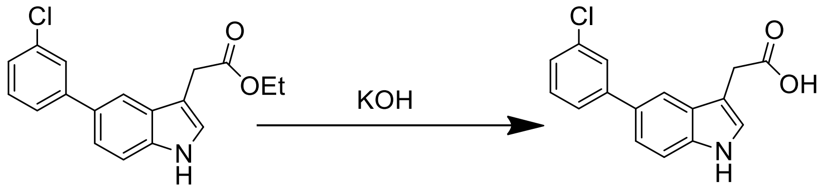

5-(3-chlorophenyl)-indole-3-acetic acid was synthesized as pale yellow solid (31 mg, 0.11 mmol, yield 90%) from the ester (38 mg, 0.12 mmol).^{1}H-NMR (400 MHz, $CDCl_3$) δ 8.13 (s, 1H), 7.76 (s, 1H), 7.61 (t, J = 1.8 Hz, 1H), 7.49 (dt, J = 7.8, 1.4 Hz, 1H), 7.40 (s, 2H), 7.34 (t, J = 7.8 Hz, 1H), 7.26-7.28 (m, 1H), 7.20 (d, J = 2.3 Hz, 1H), 3.82 (s, 2H), ^{13}C-NMR (100MHz, $CDCl_3$) δ 177.42, 144.36, 135.92, 134.54, 132.15, 129.94, 127.74, 127.54, 126.45, 125.66, 124.26, 122.15, 117.51, 111.73, 108.39, 30.98; FABMS *m/z* 286 $[M+H]^+$.
