# Supplementary material for: The auxin-inducible degron 2 technology provides sharp degradation control in yeast, mammalian cells, and mice
Source: Nat Commun. 2020 Nov 11;11:5701. doi: 10.1038/s41467-020-19532-z (PMC7659001; doi:10.1038/s41467-020-19532-z)
Supplement: Supplementary file 2 — Reporting Summary [file 41467_2020_19532_MOESM2_ESM.pdf]

nature research

Corresponding author(s): Masato Kanemaki

Last updated by author(s): Oct 9, 2020

Reporting Summary

Nature Research wishes to improve the reproducibility of the work that we publish. This form provides structure for consistency and transparency in reporting. For further information on Nature Research policies, see our Editorial Policies and the Editorial Policy Checklist.

Statistics

For all statistical analyses, confirm that the following items are present in the figure legend, table legend, main text, or Methods section.

n/a	Confirmed	
☐	☒	The exact sample size (n) for each experimental group/condition, given as a discrete number and unit of measurement
☐	☒	A statement on whether measurements were taken from distinct samples or whether the same sample was measured repeatedly
☐	☒	The statistical test(s) used AND whether they are one- or two-sided<br>*Only common tests should be described solely by name; describe more complex techniques in the Methods section.*
☒	☐	A description of all covariates tested
☒	☐	A description of any assumptions or corrections, such as tests of normality and adjustment for multiple comparisons
☐	☒	A full description of the statistical parameters including central tendency (e.g. means) or other basic estimates (e.g. regression coefficient) AND variation (e.g. standard deviation) or associated estimates of uncertainty (e.g. confidence intervals)
☐	☒	For null hypothesis testing, the test statistic (e.g. F, t, r) with confidence intervals, effect sizes, degrees of freedom and P value noted<br>*Give P values as exact values whenever suitable.*
☒	☐	For Bayesian analysis, information on the choice of priors and Markov chain Monte Carlo settings
☒	☐	For hierarchical and complex designs, identification of the appropriate level for tests and full reporting of outcomes
☒	☐	Estimates of effect sizes (e.g. Cohen's d, Pearson's r), indicating how they were calculated

Our web collection on statistics for biologists contains articles on many of the points above.

Software and code

Policy information about availability of computer code

Data collection	BD Accuri C6 flowcytometer, GE deltavision microscope
Data analysis	Graphpad Prism 6 (unpaired t-test, two-way ANOVA, Row means with SD or SEM, Nonlinear regression (curve fit) with inhibitor vs. response or one phase decay), FSC Express 4, Volocity 6.3.1, Image J, SAMtools. GSNAP, featureCounts, and R for data analysis.

For manuscripts utilizing custom algorithms or software that are central to the research but not yet described in published literature, software must be made available to editors and reviewers. We strongly encourage code deposition in a community repository (e.g. GitHub). See the Nature Research guidelines for submitting code & software for further information.

Data

Policy information about availability of data

All manuscripts must include a data availability statement. This statement should provide the following information, where applicable:

- Accession codes, unique identifiers, or web links for publicly available datasets
- A list of figures that have associated raw data
- A description of any restrictions on data availability

Transcriptome data shown in Supplementary Figure 3C are deposited in the DNA Data Bank of Japan (DDBJ) Sequencing Read Archive under the accession numbers DRA009832 and DRA010661. Other data that support the findings of this study are available from the corresponding author upon request.

Field-specific reporting

Please select the one below that is the best fit for your research. If you are not sure, read the appropriate sections before making your selection.

- [x] Life sciences
- [ ] Behavioural & social sciences
- [ ] Ecological, evolutionary & environmental sciences

For a reference copy of the document with all sections, see nature.com/documents/nr-reporting-summary-flat.pdf

Life sciences study design

All studies must disclose on these points even when the disclosure is negative.

Sample size	No statistical methods were used to predetermine sample sizes. Sample sizes were determined based on previous experience (Nishimura et al. Nature Methods, 6, 917-912, 2009; Natsume et al. Cell Reports, 15, 210-218, 2016). In case of testing degradation in a clone, we used at least two independent clones to confirm the consistency.
Data exclusions	No data was excluded from the analysis.
Replication	All experiments were reliably reproduced. Each experiment was performed independently at least two times, but usually more than three times.
Randomization	No randomization was performed for cell analyses because of complication of the experiments. Mice were paired based on gender, age and weight.
Blinding	Blinding was not performed in this study because of complication of the experiments.

Reporting for specific materials, systems and methods

We require information from authors about some types of materials, experimental systems and methods used in many studies. Here, indicate whether each material, system or method listed is relevant to your study. If you are not sure if a list item applies to your research, read the appropriate section before selecting a response.

Materials & experimental systems

n/a	Involved in the study	
[ ]	[x]	Antibodies
[ ]	[x]	Eukaryotic cell lines
[x]	[ ]	Palaeontology and archaeology
[ ]	[x]	Animals and other organisms
[x]	[ ]	Human research participants
[x]	[ ]	Clinical data
[x]	[ ]	Dual use research of concern

Methods

n/a	Involved in the study	
[x]	[ ]	ChIP-seq
[ ]	[x]	Flow cytometry
[x]	[ ]	MRI-based neuroimaging

Antibodies

Antibodies used	For protein detection, the following commercially available antibodies were used. Primary antibodies: anti-mAID (MBL, #M214-3), anti-OsTIR1 (MBL, #PD048), anti-V5 (Invitrogen, #R960-25), anti-DHC1 (SantaCruz, #sc-9115), anti-SMC2 (Bethyl, #A300-058A-T), anti-CTCF (Bethyl, #A300-543-T), anti-POLR2A (Abcom, #ab817), anti-BRD4 (GeneTex, #GTX130586), anti-TOP2A (MBL, #M042-3S), anti-alpha-tubulin (MBL, #M175-3). Secondary antibodies: anti-rabbit IgG HRP (GE Healthcare, #NA934), anti-mouse IgG HRP (SantaCruz, #PI-2000), anti-rabbit IgG StarBright Blue 700 (Bio-Rad, #12004161), Alexa Fluor 647 anti-mouse IgG (ThermoFisher, #A-21236).
Validation	All used primary antibodies were validated by the manufactures and previous publications. References can be found at the manufactures' web site.<br>https://ruo.mbl.co.jp/bio/dtl/A/?pcd=PD048<br>https://www.scbt.com/p/dynein-hc-antibody-r-325?productCanUrl=dynein-hc-antibody-r-325&_requestid=682743<br>https://www.bethyl.com/product/A300-058A/SMC2+Antibody<br>https://www.bethyl.com/product/A300-543A/CTCF+Antibody<br>https://www.abcam.com/rna-polymerase-ii-ctd-repeat-ysptsps-antibody-8wg16-chip-grade-ab817.html<br>https://www.genetex.com/Product/Detail/BRD4-antibody/GTX130586<br>https://ruo.mbl.co.jp/bio/e/dtl/A/?pcd=M042-3<br>https://ruo.mbl.co.jp/bio/e/dtl/A/?pcd=M175-3<br>https://www.sigmaaldrich.com/catalog/product/sigma/t4026?lang=ja&region=JP<br>https://www.bio-rad.com/en-jp/sku/12004165-hfab-rhodamine-anti-tubulin-primary-antibody-200-ul?ID=12004165

Eukaryotic cell lines

Policy information about cell lines

Cell line source(s)	HCT116 and HEK293 cell lines were originally obtained from ATCC.
Authentication	The original HCT116 and HECk293 were authenticated by ATCC. Other HCT116 derivative cell lines were not authenticated. However, all HCT116 cell lines used in this study were frequently checked their morphology, growth, and karyotype.
Mycoplasma contamination	The original HCT116 line was tested, but other resultant clones were not tested for mycoplasma contamination. However, we did not observe any phenotypes related to mycoplasma infection.
Commonly misidentified lines (See ICLAC register)	No commonly misidentified cell lines were used.

Animals and other organisms

Policy information about studies involving animals; ARRIVE guidelines recommended for reporting animal research

Laboratory animals	To obtain hippocampal neurons (Figure 2), pregnant female C57BL/6J mice (10- or 11-week old) were obtained from Japan SLC. Nude mice used for xenograft assay in Figure 5 were Balb/c-nu female mice (7-week old) weighing 16 to 20 g and were obtained from Charles River Japan. To generate transgenic (TG) mice (Figure 6), female B6C3F1 (C57BL/6N X C3H/HeN) (4- or 5-week old) were obtained from CLEA Japan.
Wild animals	None.
Field-collected samples	None.
Ethics oversight	All protocols and procedures involving the care and use of animals were reviewed and approved by the Institutional Animal Care and Use Committee (IACUC) of National Institute of Genetics and The University of Tokyo prior to conduct. Throughout the study, the care and use of animals were conducted in accordance with the guidelines and regulations set by the Ministry of Education, Culture, Sports, Science and Technology (MEXT), the Ministry of the Environment, and the Science Council of Japan.

Note that full information on the approval of the study protocol must also be provided in the manuscript.

Flow Cytometry

Plots

Confirm that:

- [x] The axis labels state the marker and fluorochrome used (e.g. CD4-FITC).
- [x] The axis scales are clearly visible. Include numbers along axes only for bottom left plot of group (a 'group' is an analysis of identical markers).
- [x] All plots are contour plots with outliers or pseudocolor plots.
- [x] A numerical value for number of cells or percentage (with statistics) is provided.

Methodology

Sample preparation	For detecting EGFP and Clover signals after ligand treatment, cells were trypsinized and fixed in 4% methanol-free paraformaldehyde phosphate buffer (FUJIFILM Wako Pure Chemical Corporation) at 4oC overnight. Fixed cells were washed and resuspended in PBS containing 1% BSA. For measuring the DNA signal after ligand treatment, cells were trypsinized and fixed in 70% EtOH. Fixed cells were washed, resuspended in PBS containing 1% BSA, 50 µg/ml of RNase A, and 40 µg/ml of propidium iodide, and incubated at 37oC for 30 min. 10,000 cells were analyzed from each sample.
Instrument	BD Accuri C6
Software	FSC4 Express
Cell population abundance	No cell sorting was done in this study.
Gating strategy	Cells were gated with SSC and FSC for analyses.

- [x] Tick this box to confirm that a figure exemplifying the gating strategy is provided in the Supplementary Information.
