# Supplementary material for: The auxin-inducible degron 2 technology provides sharp degradation control in yeast, mammalian cells, and mice
Source: Nat Commun. 2020 Nov 11;11:5701. doi: 10.1038/s41467-020-19532-z (PMC7659001; doi:10.1038/s41467-020-19532-z)
Supplement: Supplementary file 3 — Peer Review File [file 41467_2020_19532_MOESM3_ESM.pdf]

Editorial Note: This manuscript has been previously reviewed at another journal that is not operating a transparent peer review scheme. This document only contains reviewer comments and rebuttal letters for versions considered at *Nature Communications*. Mentions of prior referee reports have been redacted.
